# Association between life satisfaction and health behaviours among older adults: a systematic review and meta-analysis

**DOI:** 10.1186/s12966-026-01877-1

**Published:** 2026-01-26

**Authors:** Chiedozie James Alumona, David R. Scott, Toyin Aladejebi, Michael E. Kalu, Ogochukwu Kelechi Onyeso, Adesola C. Odole, Laura Vogelsang, Jerome Singleton, Oluwagbohunmi Adetunji Awosoga

**Affiliations:** 1 Faculty of Health Sciences, University of Lethbridge, Lethbridge, Alberta Canada; 2 Emerging Researchers and Professionals in Ageing-African Network, Abuja, Nigeria; 3 Department of Physiotherapy, College of Basic Medical Sciences, Chrisland University, Abeokuta, Ogun Nigeria; 4 University of Lethbridge Library, Lethbridge, Alberta Canada; 5 School of Kinesiology and Health Sciences, Faculty of Health, York University, Toronto, Ontario Canada; 6 Department of Physiotherapy, Faculty of Clinical Sciences, College of Medicine, University of Ibadan, Ibadan, Oyo Nigeria; 7 School of Health and Human Performance, Faculty of Health, Dalhousie University, Halifax, Nova Scotia Canada

**Keywords:** Well-being, Healthy ageing, Lifestyle, Quality of life, Sustainable development goal

## Abstract

**Background:**

Life satisfaction is a key indicator of quality of life among older adults. This systematic review and meta-analysis synthesised evidence on the association between life satisfaction and health behaviours such as smoking status, alcohol use, physical activity, diet/nutrition, and sleep among older adults aged 60 years and older.

**Methods:**

The review was conducted and reported following the PRISMA guidelines. We searched the electronic databases MEDLINE, APA PsycINFO, Web of Science, CINAHL, and Global Health from inception to 10 January 2025 for observational studies reporting an association between life satisfaction and health behaviours. Two independent reviewers completed article screening, data extraction, and risk of bias assessment. The result was summarised through a narrative synthesis, and meta-analysis was completed using CMA (version 4).

**Results:**

The 56 included studies were conducted across 22 countries between 1990 and 2025. The pooled mean age and female proportion were 70.59 years (95% CI: 68.98, 72.21) and 58.0% (95% CI: 55.1, 60.7), respectively. Narrative synthesis showed that most studies found quality sleep and/or 7–8 h of sleep (77.3%), a higher physical activity level (69.1%), and a regular intake of fruit and vegetables and/or a balanced diet regularly (52.9%) were significantly associated with higher life satisfaction. Smoking and alcohol use were associated with lower life satisfaction in 33.3% and 15.8% of the analysis, respectively. The meta-analysis showed that higher physical activity levels (*r* = 0.12, *p* = 0.003) were associated with higher life satisfaction. Only four studies on physical activity met the criteria for meta-analysis, and no studies on other health behaviours did.

**Conclusions:**

Quality sleep and/or 7–8 h of sleep, a higher physical activity level, and regularly eating fruit and vegetables and/or a balanced diet are associated with higher life satisfaction. The review provides evidence for policymakers, healthcare workers, caregivers, and society to encourage healthy behaviours that foster healthy ageing. Future studies should use standardised instruments to assess health behaviours and life satisfaction, facilitating cross-study comparisons and the meta-synthesis of research findings.

**Systematic review registration:**

PROSPERO (CRD42023441386).

**Supplementary Information:**

The online version contains supplementary material available at 10.1186/s12966-026-01877-1.

## Background

Ageing is a natural and inevitable process that affects people of all races, cultures, social classes, and geographical locations [1, 2]. In 2017, 13% of the global population was aged 60 and over, and this figure is expected to rise to 21% by 2050 [3]. The World Health Organisation [4] also reported that the number of older adults will increase from 1 billion in 2020 to 2.1 billion in 2050. Given the projected increase in the ageing population, various stakeholders, including governments, policymakers, health practitioners, and researchers, are keenly interested in promoting the health and quality of life of older adults.

Life satisfaction is an indicator of the quality of life [5] and predicts morbidity and mortality among older adults [6]. It is an evaluative component of subjective well-being through which individuals measure the quality of their lives according to their perspectives [7, 8]. It is a judgment that reflects the difference between individuals’ present conditions and the ideal standard they set for themselves [8]. The narrower the gap between individuals’ current state and aspirations, the higher their life satisfaction.

The determinants of life satisfaction have been grouped into demographic and socioeconomic factors, physical and mental health, social support, and health behaviours [9, 10]. Health behaviours are everyday actions that influence health and well-being, such as dietary choices, physical activity, and substance use. Recent systematic reviews examined mainly the association between life satisfaction and psychophysical, socioeconomic, and demographic characteristics among older adults [11–13], leaving a gap in the literature on the influence of health behaviours. Existing reviews on health behaviours focused on well-being indicators, including anxiety, depression, emotional distress, or functional quality of life [14–16], which are conceptually distinct from life satisfaction. To our knowledge, no review has synthesised evidence of the association between health behaviours and life satisfaction. Comprehending how health behaviours contribute to individuals’ life satisfaction in old age is crucial to improving well-being and guiding care and policy development [9, 10]. The current review adds to the existing literature by synthesising the association between life satisfaction and five key health behaviours: smoking, alcohol use, physical activity, diet/nutrition, and sleep, which have been reported in primary studies [17–19].

Guided by the biopsychosocial framework [20], we conceptualised the influence of health behaviours on life satisfaction as occurring through multiple pathways, including biological, psychological, and social means. From the biological perspective, health behaviours impact physical health, functional ability, and energy levels [21]. Psychologically, they can affect mood, self-image, self-esteem, and stress management [22]. Socially, they influence the engagement in meaningful activities and social connectedness [23, 24]. These pathways suggest that health behaviours are vital targets for interventions aimed at enhancing life satisfaction.

The study was anchored on the United Nations’ Sustainable Development Goal (SDG) three: to ensure healthy lives and promote well-being for all, including older adults [25]. Promoting healthy behaviours among older adults is the bedrock of preventing and reducing mortality rates from non-communicable diseases and enhancing well-being [26, 27]. Moreover, the inclusion of a campaign against substance abuse, including narcotic abuse, and harmful alcohol use, among the strategies to achieve the SDG goal three suggests the importance of behaviours to enhance a healthy ageing experience.

Therefore, we conducted a systematic review and meta-analysis of the association between health behaviours and life satisfaction among older adults. The review aimed to describe the direction and strength of the association between health behaviours and life satisfaction among community-dwelling older adults. Since health behaviours are modifiable through personal choices and targeted policy interventions, the outcome of this review will benefit ageing research, clinical practice, and policymaking regarding evidence-based practice.

## Methods

### Protocol and registration

The protocol was registered with the International Prospective Register of Systematic Reviews (PROSPERO, CRD42023441386) and also published [17]. The review was conducted and reported according to the updated Preferred Reporting Items for Systematic Reviews and Meta-Analyses (PRISMA-2020) [28] and the Meta-analysis of Observational Studies in Epidemiology (MOOSE) [29] guidelines. The PRISMA-2020 checklist is given in Supplementary File 1.

### Population, exposure, outcome, and timeline (PEOT) criteria

The *population* was community-dwelling older adults aged 60 years and over. The *exposure* was behavioural factors: smoking, alcohol use, physical activity, diet/nutrition, and sleep, which have been widely researched [18, 19]. Smoking was defined as a habit (yes, no, current, former, or never) [10, 18], while alcohol use was operationalised as either a habit or a level of consumption [9, 30]. Physical activity was mainly evaluated by the level of activity engaged in [31, 32]. For older adults, good sleep refers to getting 7 to 8 h of quality sleep with minimal disturbance [19, 33]. A healthy diet was operationalised as eating fruit and vegetables or a balanced diet regularly [34, 35]. The *outcome* was life satisfaction assessed with self-report measures, including satisfaction with life scale, life satisfaction index, and a single-item question [36–38]. The review *timeline* was from the inception of each database to 10 January 2025.

### Eligibility criteria

Studies were included if (1) they were observational studies describing the association between any of the health behaviours and life satisfaction, (2) conducted among apparently healthy community-dwelling older adults with a mean age of ≥ 60 years, (3) written in English language, (4) peer-reviewed, and (5) published on or before 10 January 2025. Studies were included in the meta-analysis if they reported zero-order associations between life satisfaction and health behaviour, as adjusted effect sizes would introduce bias due to differing covariate adjustment, limiting comparability across studies. Studies were excluded if (1) they were qualitative, (2) conducted among older adults with specific disease conditions, including stroke, dementia, diabetes, and Alzheimer’s disease, and (3) residing in institutions such as nursing homes and long-term care facilities.

### Information sources

Following the recommendation of an optimal database combination [39], we searched the MEDLINE, APA PsycINFO, Web of Science, CINAHL, and Global Health databases from inception to 10 January 2025. We also hand-searched and reviewed articles identified through the reference lists of all included articles.

### Search strategy

**Fig. 1:**
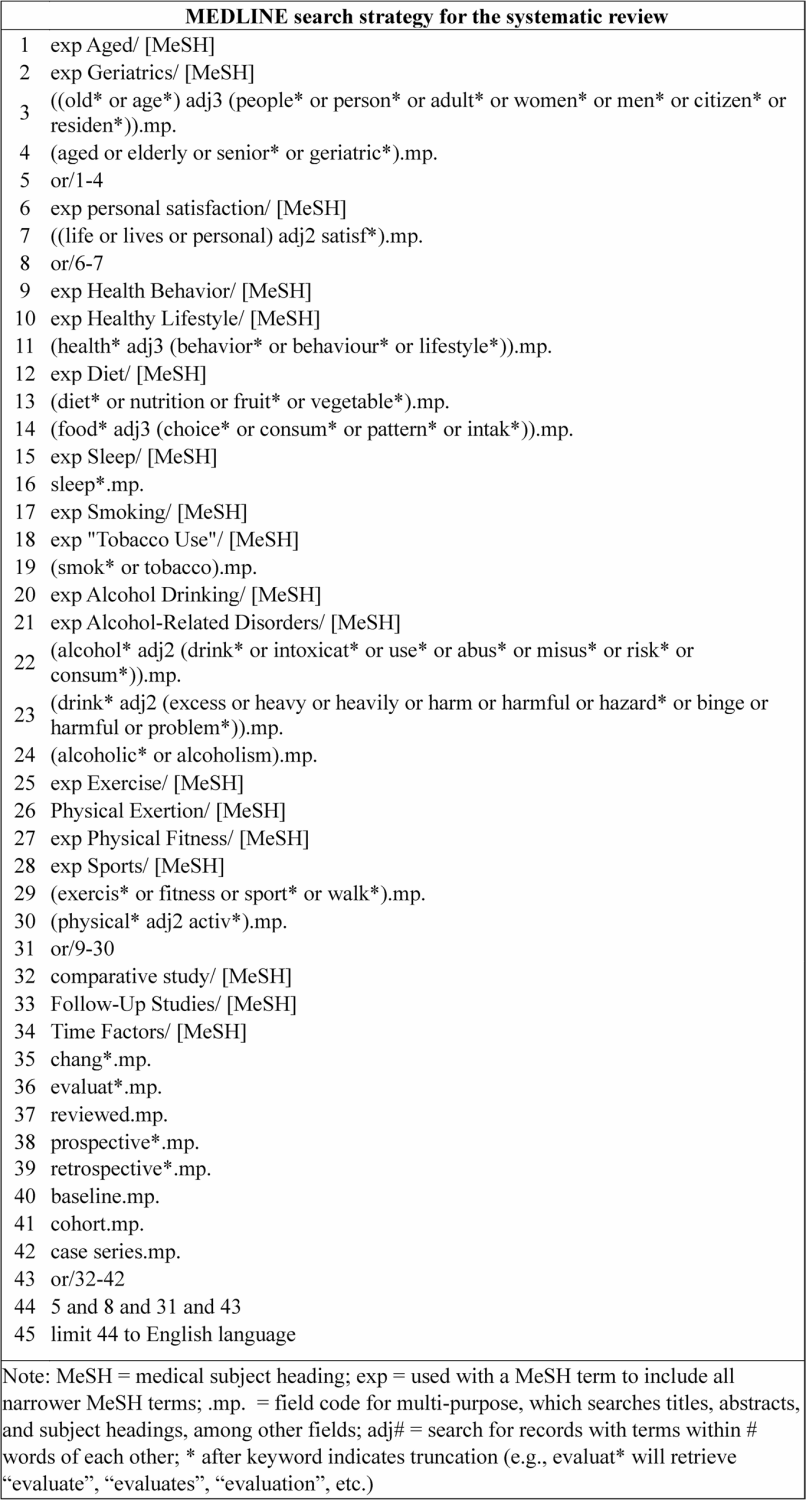
MEDLINE search strategy for the systematic review

Search terms were identified through consultations between the primary investigator (ACJ), content experts (OAA, ACO, LV and JS), and the librarian (DRS) and a review of the titles and abstracts of nine seed articles [9, 10, 18, 19, 34, 40–43] gathered by the primary investigator [17]. Elements of search strings developed for previously published reviews also informed the search strategy [11, 44–48]. The search string was first developed for MEDLINE (Fig. 1) and then adapted for the other four databases (Supplementary File 2).

When possible, subject headings from controlled vocabularies (e.g., MeSH) were used in the search. To increase sensitivity, concepts were also entered in the search string as keywords, with truncation (e.g., diet*) and proximity operators (e.g., adj3) used when appropriate. Boolean operators connected subject headings and keywords as shown in Fig. 1. No limits were placed on publication dates, though results were limited to studies written in English.

### Selection process

The retrieved articles’ bibliographic information (e.g., title, abstract, authors, publication information, subject headings) was imported into EndNote 20 for deduplication [39]. After deduplication, all the remaining citations were transferred to Covidence, a systematic review management tool [49] for title/abstract and full-text screening. Covidence randomly assigned articles to two independent screeners from the authorship list and conflict resolution to a third independent reviewer (CJA or MEK).

### Data collection process

Three reviewers (CJA, TA, OKO) conducted a pilot data extraction of five articles using a customised Microsoft Excel spreadsheet. The goal was to identify and discuss potential ambiguities in the data extraction form and to calibrate the understanding of the extraction criteria, ensuring uniformity in their approach. Thereafter, a pair of the reviewers completed the data extraction and reviewed the reference lists of the included articles.

### Data items

We extracted the following data from each article: first author’s surname, year of publication, country of publication, title of the study, study design (e.g., cross-sectional or longitudinal), sample size, name of secondary dataset, measures of health behaviours (smoking, alcohol drinking, physical activity, diet/nutrition, and sleep) and life satisfaction, and descriptive summary of age, sex, health behaviours and life satisfaction. Additionally, we collected inferential statistical results, including correlation and regression coefficients, other effect sizes, effect directions, confidence intervals, and *p*-values.

### Risk of bias assessment

Two reviewers (TA, OKO) independently assessed the risk of bias (ROB) using the Joanna Briggs Institute (JBI) critical appraisal tool for analytical cross-sectional studies [50], and a third reviewer (CJA) resolved the disagreements. The 8-item JBI tool provides clear instructions and guidelines for evaluating a study’s clarity of inclusion criteria, descriptions of participants and settings, measures of exposure, conditions, and outcomes, strategies for identifying and dealing with confounding factors, and appropriateness of statistical analysis used [51]. Each item was rated as “Yes = 1,” “Unclear = 0,” or “No = 0,” with a total score ranging from 0 to 8 and categorised as High (0–3), Moderate (4–5), or Low (6–8).

### Data synthesis methods

#### Narrative synthesis

The units of synthesis were health behaviours, types of inferential statistics (bivariate and multivariate analysis), and study designs (cross-sectional and longitudinal). A narrative synthesis was completed to illustrate the direction of effects across all included studies. The synthesis for each health behaviour was reported under the headings of bivariate analyses (including simple linear regression, Pearson correlation, and tests of differences, such as t-test and analysis of variance) and multivariate analyses.

#### Meta-analysis

The meta-analysis was completed using the Comprehensive Meta-Analysis (CMA, version 4) software [52]. The pooled effect size was computed using a random-effects model, which assumes that the studies are a random sample from a universe of potential studies and allows statistical inference to be made on studies not included in the analysis [53, 54]. The pooled effect size was Fisher’s z-transformed correlation coefficient and reported with its 95% confidence interval and *p*-value [55]. The CMA software weights studies by inverse variance and calculates the weighted average by aggregating the weights of the individual studies. The software generated the forest and funnel plots for the pooled estimate and the publication bias, respectively.

Three indicators of heterogeneity: *Q*-statistic, *I*^*2*^ statistic and Tau-squared (Tau^*2*^) were used to assess the heterogeneity of the included studies [56]. A *Q*-statistic tested the null hypothesis that the included studies have the same effect size. If all the studies have the same effect size, the *p*-value will be greater than or equal to the criterion alpha of 0.1. An *I*^*2*^ statistic shows the percentage variance in the observed effect that is accounted for by the true effect rather than sampling error, while Tau^*2*^ shows the variance of the true effects. Finally, a 95% Prediction Interval was computed to determine the effect size range that 95% of all studies comparable to those in the analysis will fall [56]. An *I²* statistic of 50% or less was set as the threshold for proceeding with the meta-analysis [57]. To minimise heterogeneity, the meta-analysis was conducted across studies using the same instrument, with a plan to perform a subgroup analysis based on publication date if higher heterogeneity was observed.

A sensitivity analysis using a “one-study-removed” approach was computed to determine if the basic conclusion of the meta-analysis would change [58]. Publication bias was assessed using two indicators: the funnel plot and Egger’s regression intercept [52].

## Results

### Study characteristics

**Fig. 2:**
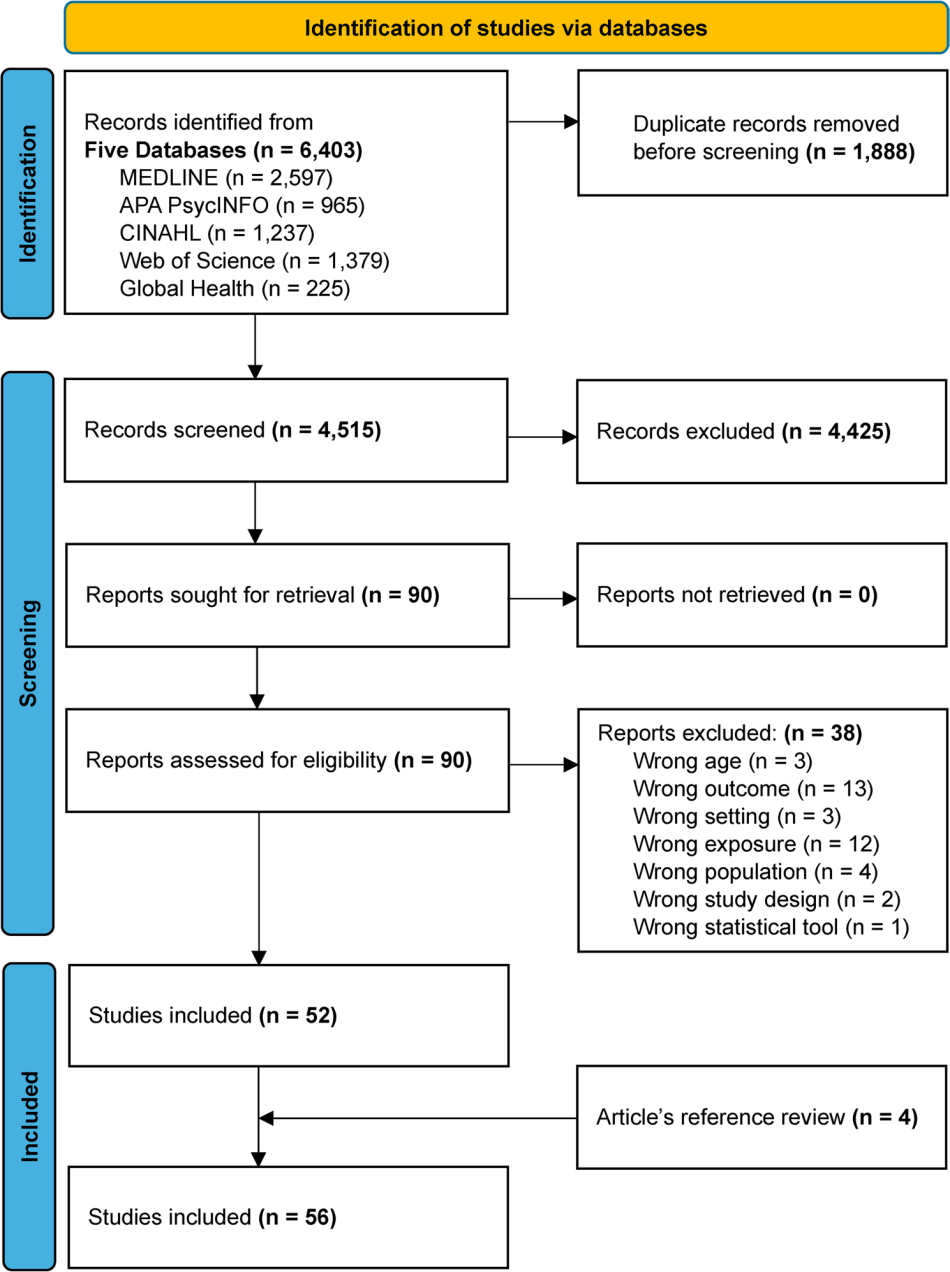
PRISMA flowchart

The search across five databases yielded 6,403 citations, of which 1,888 duplicates were removed (Fig. 2). The team screened 4,515 titles and abstracts, excluding 4,425 studies that did not meet the inclusion criteria. The full texts of the remaining 90 articles were retrieved and screened, yielding 52 articles for data extraction. Four additional articles were found through a review of the references of the included articles, bringing the total to 56 articles.

**Fig. 3:**
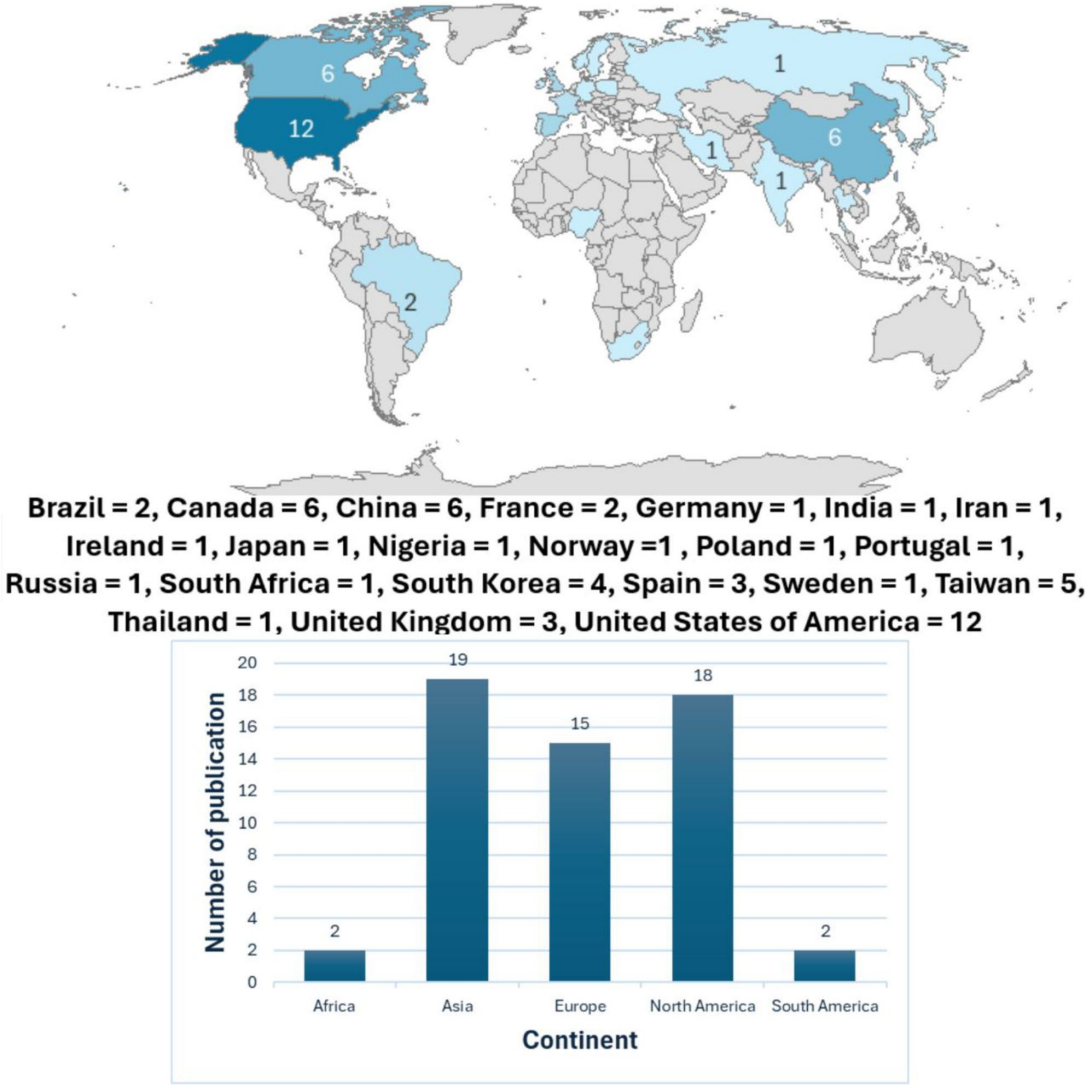
Countries where the included studies were conducted

The studies were conducted across 22 countries between 1990 and 2025 (Fig. 3). The studies’ characteristics (Table 1 and Supplementary File 3) showed that 38 (67.9%) employed cross-sectional analyses, 24 (42.9%) were published between 2011 and 2020, 34 (60.7%) utilised secondary datasets, 24 (42.9%) assessed life satisfaction using a single-item question, and 41 (73.2%) focused on a single health behaviour. The pooled mean age and proportion of women were 70.59 years (95% CI: 68.98, 72.21) and 58.0% (95% CI: 55.1, 60.7), respectively.

**Table 1:** Study characteristics

Characteristics	Frequency	Percentage
Study design		
Cross-sectional	38	67.9
Longitudinal	12	21.4
Both cross-sectional and longitudinal	6	10.7
Year of publication		
1990–2010	10	17.9
2011–2020	24	42.9
2021–2025	22	39.2
Secondary dataset		
Yes	34	60.7
No	22	39.3
Life satisfaction measure		
Satisfaction With Life Scale	18	32.1
Life Satisfaction Index	7	12.5
Single-item question	24	42.9
Others	7	12.5
Number of health behaviours		
Single	41	73.2
Multiple	15	26.8

The summary of the narrative synthesis (Table 2) showed that 77.3%, 69.1%, and 52.9% of the unique analyses found an association between higher life satisfaction and good sleep, physical activity level, and healthy diet/nutrition, respectively. Smoking and alcohol use were associated with lower life satisfaction in 33.3% and 15.8% of the analysis, respectively. Supplementary File 4 shows the summary of results from the 56 included studies.

**Table 2:** Summary of the narrative synthesis of the association between health behaviours and life satisfaction

Result	Bivariate analysis f (%)		Multivariate analysis f (%)		Total f (%)
	Cross-sectional	Longitudinal	Cross-sectional	Longitudinal	
Smoking					
Smoking → significantly lower life satisfaction	1 (33.3)	1 (50.0)	1 (16.7)	3 (42.9)	6 (33.3)
Smoking → significantly higher life satisfaction	-	-	-	-	-
Result was not statistically significant	2 (66.7)	1 (50.0)	5 (83.3)	4 (57.1)	12 (66.7)
Alcohol drinking					
Alcohol use → significantly lower life satisfaction	1 (25.0)	-	1 (14.3)	1 (14.3)	3 (15.8)
Alcohol use → significantly higher life satisfaction	-	-	1 (14.3)	-	1 (5.3)
Result was not statistically significant	3 (75.0)	1 (100.0)	5 (71.4)	6 (85.7)	15 (78.9)
Physical activity					
Higher physical activity level → significantly higher life satisfaction	16 (69.6)	4 (80.0)	8 (57.1)	10 (76.9)	38 (69.1)
Lower physical activity level → significantly higher life satisfaction	1 (4.3)	-	-	-	1 (1.8)
Result was not statistically significant	6 (26.1)	1 (20.0)	6 (42.9)	3 (23.1)	16 (29.1)
Diet/Nutrition					
Healthy diet/nutrition → significantly higher life satisfaction	5 (71.4)	-	2 (33.3)	1 (100.0)	9 (52.9)
Unhealthy diet/nutrition → significantly higher life satisfaction	-	-	-	-	-
Result was not statistically significant	2 (28.6)	2 (100.0)	4 (66.7)	-	8 (47.1)
Sleep					
Good sleep → significantly higher life satisfaction	8 (80.0)	1 (100.0)	5 (71.4)	3 (75.0)	17 (77.3)
Poor sleep → significantly higher life satisfaction	-	-	-	-	-
Result was not statistically significant	2 (20.0)	-	2 (28.6)	1 (25.0)	5 (22.7)

Good sleep refers to getting 7 to 8 h of quality sleep with minimal disturbance. A healthy diet was operationalised as eating fruit and vegetables or a balanced diet regularly. NB: The total number of studies included was 55, but some studies have different results based on analysis types (bivariate vs. multivariate), study design (cross-sectional vs. longitudinal), subgroups (men vs. women), years of follow-ups (e.g. three vs. four), and type of longitudinal life satisfaction (e.g. incident vs. persistent)

### Smoking

Eighteen uniques analyses across thirteen studies examined the association between smoking habits and life satisfaction (Table 2). Six analyses reported that smoking was significantly associated with lower life satisfaction, while 12 analyses found no significant association. Among the analyses showing significant results, two reported bivariate associations, and four were multivariate analyses that retained significance after adjusting for covariates. No study reported that smoking was associated with higher life satisfaction. The following paragraphs provide a detailed synthesis and citations organised by analytical approach and study design.

#### Bivariate analysis

A cross-sectional bivariate analysis found that the prevalence of being satisfied with life was significantly higher among older adults who never smoked [59]. However, two cross-sectional bivariate analyses found no significant association between smoking status and life satisfaction [18, 60]. Similarly, no significant longitudinal association was found between smoking and incident life satisfaction among older men after four years of follow-up [30], though smokers had higher odds of persistent lower life satisfaction [30].

#### Multivariate analysis

A cross-sectional multivariate analysis found that being a smoker significantly predicted lower life satisfaction [32]. Two cross-sectional analyses reported the same effect direction, but the coefficients were not statistically significant [10, 61]. Four cross-sectional studies found smokers to have slightly higher life satisfaction, but the coefficients were not statistically significant [9, 10, 18, 62]. The longitudinal multivariate analyses showed that smoking was a significant predictor of lower life satisfaction after the follow-up periods of four [30, 63] and 16 years [35]. However, four longitudinal studies found no significant association between smoking and life satisfaction after two [61], five [31], and seven years of follow-up [32, 64].

### Alcohol use

Nineteen analyses from 14 studies explored the association between alcohol use and life satisfaction (Table 2). Three analyses reported that alcohol use was significantly associated with lower life satisfaction, while 15 analyses found no significant association. In contrast, one analysis reported that alcohol use was associated with higher life satisfaction. Among the analyses showing significant results, one was bivariate, and two were multivariates analyses.

#### Bivariate analysis

A cross-sectional study found that heavy alcohol drinkers had significantly lower life satisfaction than non-heavy drinkers [65], while three studies found no significant association [18, 59, 60]. There was no significant longitudinal influence of alcohol dependence on life satisfaction after four years [30].

#### Multivariate analysis

Di Gessa and Zaninotto [66] and Jung et al. [10] reported high alcohol use to be significantly associated with lower life satisfaction in older people and higher life satisfaction in men, respectively, while five other studies found no significant influence of alcohol use [9, 10, 18, 32, 61]. A longitudinal analysis showed that increased alcohol drinking over 6 months significantly decreased life satisfaction [66]. However, there was no significant effect of alcohol use on life satisfaction after follow-up periods of two [61], five [31], seven [32, 64], eight [67], and 16 years [35].

### Physical activity

Fifty-five analyses from 37 studies examined the association between physical activity and life satisfaction (Table 2). Thirty-eight analyses reported that higher physical activity levels were significantly associated with higher life satisfaction, while 16 analyses found no significant association. In contrast, one analysis reported that lower physical activity was associated with higher life satisfaction. Among the analyses showing significant results, 21 were bivariate, and 18 retained significance after adjusting for covariates.

#### Bivariate analysis

Sixteen cross-sectional bivariate analyses found that higher physical activity was significantly associated with higher life satisfaction [18, 37, 41, 43, 59, 60, 67–76], while Bourque et al. [77] reported a significant association between lower physical activity and higher life satisfaction. Six cross-sectional analyses reported a non-significant association between higher physical activity and higher life satisfaction [38, 43, 78–81]. Four bivariate longitudinal analyses showed a significant association between higher physical activity and greater life satisfaction after three [82], four [30], seven [36], and eight years of follow-up [67]. However, an 18-month longitudinal analysis found no significant association between higher physical activity and higher life satisfaction [43].

#### Multivariate analysis

Eight multivariate cross-sectional analyses showed that higher physical activity significantly predicted greater life satisfaction [18, 37, 59, 66, 68, 73, 75, 83]. However, six cross-sectional analyses reported a non-significant influence of physical activity on life satisfaction [10, 32, 41, 61, 77, 84]. The longitudinal multivariate analyses revealed that higher physical activity contributed significantly to higher life satisfaction after the follow-up periods of six months [66], one year [85], three [82], four [30, 86], six [87], seven [36, 64], eight [67], and 16 years [35]. No significant association was reported by Shojima et al. [61], Gureje et al. [31], and Peng et al. [32] after two-, five-, and seven-year follow-up, respectively.

#### Meta-analysis

**Fig. 4:**
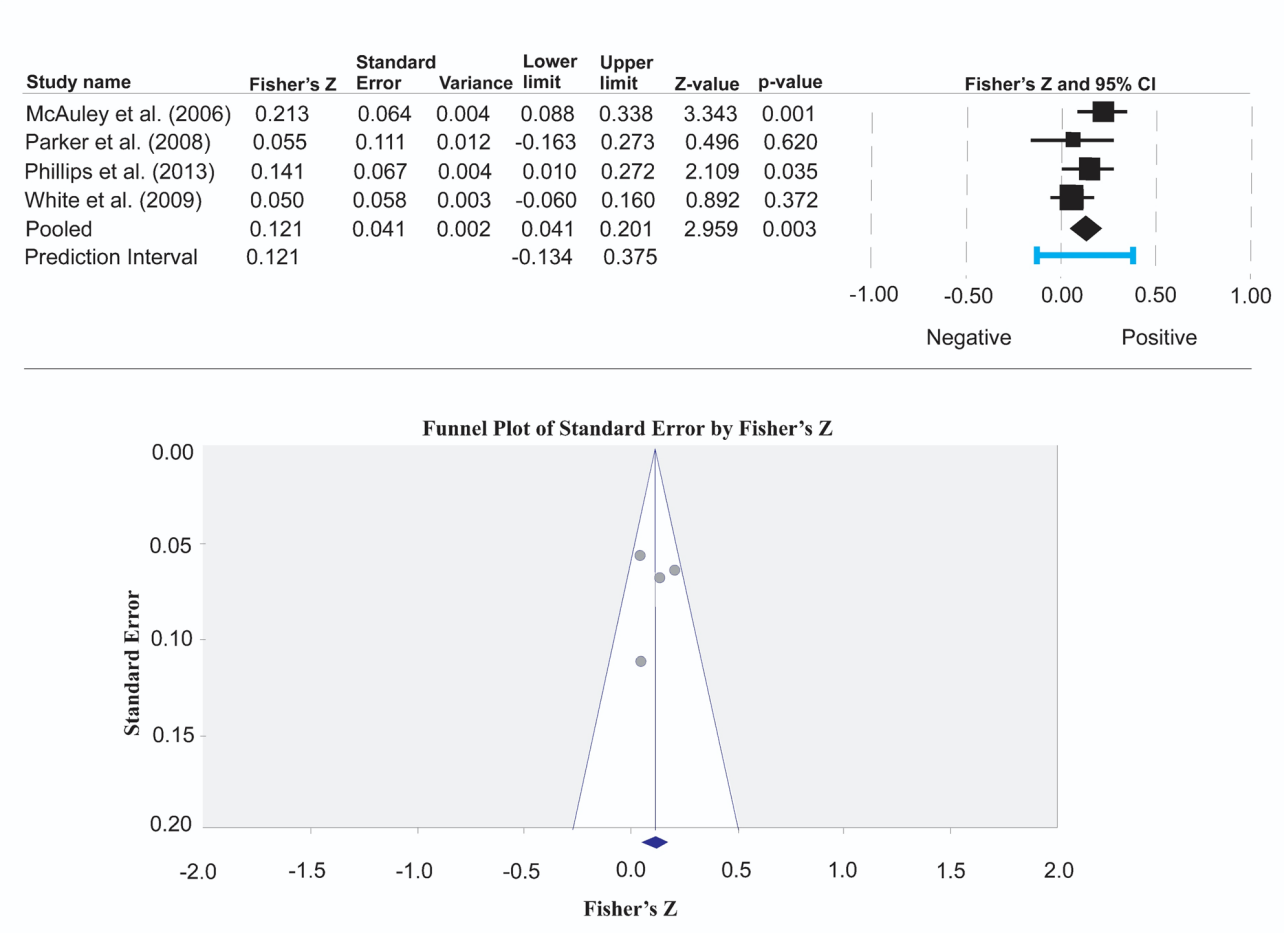
Meta-analysis of the association between physical activity and life satisfaction

Four cross-sectional studies [38, 43, 71, 80] that examined the bivariate association between physical activity and life satisfaction using the same instruments, Physical Activity Scale for the Elderly and the Satisfaction With Life Scale, respectively, were included in the meta-analysis (Fig. 4). We did not complete a separate meta-analysis of other instruments because not more than two studies assessed both physical activity and life satisfaction with the same tools.

The result shows that the pooled correlation coefficient between physical activity and life satisfaction is significant, *r* = 0.12 (95% CI = 0.04, 0.20), *p* = 0.003. The Q-test for heterogeneity shows that the true effect size is the same across all studies (*Q* = 4.13, *df* = 3, *p* = 0.248). The *I*^*2*^ statistic shows that 27.4% of the variance in the observed effects is accounted for by the true effects rather than sampling error (*I*^*2*^ statistic = 27.4), and the variance of true effect sizes (Tau^2^) is 0.002. The prediction interval is -0.13 to 0.38, reflecting the range in which the true effect size falls in 95% of comparable studies. Sensitivity analysis by removing one of the studies [71] with the largest effect size showed that the basic conclusions did not change, with a pooled effect size r = 0.08 (95% CI = 0.01, 0.16), *p* = 0.037. There was no statistical evidence of publication bias; Egger’s test = -0.325, 95% CI = -14.20, 13.56, *p* = 0.929.

### Diet/nutrition

Seventeen analyses across 12 studies investigated the association between diet/nutrition and life satisfaction (Table 2). Nine analyses reported that a healthy diet was significantly associated with higher life satisfaction, while eight analyses found no significant association. Among the analyses showing significant results, five were bivariate, and three retained the significant association after covariate adjustment in multivariate analyses. No analyses reported that an unhealthy diet was associated with higher life satisfaction.

#### Bivariate analysis

Five cross-sectional bivariate analyses found that being in a healthy food cluster [34] and a higher fruit and vegetable consumption [5, 18, 88, 89] were significantly associated with greater life satisfaction, while only two studies reported a non-significant association [60, 90]. A five-year and four-year longitudinal bivariate analysis reported no significant association between improved diet and increased life satisfaction [91] and between fruit/vegetable intake and life satisfaction [30], respectively.

#### Multivariate analysis

In a cross-sectional analysis, Jung et al. [10] reported that a higher degree of nutritional diet significantly predicted a higher life satisfaction among both older men and women, while Zaragoza-Marti et al. [92] found that a Mediterranean diet was associated with higher life satisfaction among older women, but not among older men. Three cross-sectional multivariate analyses reported a non-significant association between a fruit and vegetable diet and higher life satisfaction [18, 88, 90]. A 16-year longitudinal multivariate analysis showed that fruit and vegetable intake significantly influenced higher life satisfaction [35].

### Sleep

Twenty-two analyses across fourteen studies explored the association between sleep and life satisfaction. Seventeen analyses reported that good sleep was significantly associated with higher life satisfaction, while five analyses found no significant association. Among the analyses showing significant results, nine were bivariate and eight were multivariate analyses No analyses reported that poor sleep was associated with higher life satisfaction.

#### Bivariate analysis

Most cross-sectional bivariate analyses reported that quality sleep [19, 93–95], less sleep disturbance [33, 37], normal or longer sleep duration [19, 96, 97] were significantly associated with higher life satisfaction, while only two cross-sectional studies found a non-significant association [74, 98]. A seven-year longitudinal bivariate analysis found that longer sleep duration was associated with higher life satisfaction [96].

#### Multivariate analysis

The cross-sectional multivariate analyses reported that quality sleep [19, 93], less sleep disturbance [33], and longer sleep duration [19, 66, 97] were significantly associated with higher life satisfaction [19, 33, 66, 93, 97]. However, two cross-sectional analyses found no significant association between sleep and life satisfaction [32, 37]. The longitudinal multivariate analyses showed that longer sleep duration was significantly associated with increased life satisfaction after six months of follow-up [66]. Similarly, quality sleep was associated with higher life satisfaction after three and four years [99] and seven years of follow-up [64]. A seven-year longitudinal multivariate analysis reported no significant influence of sleep duration on life satisfaction [32].

### Risk of bias

**Fig. 5:**
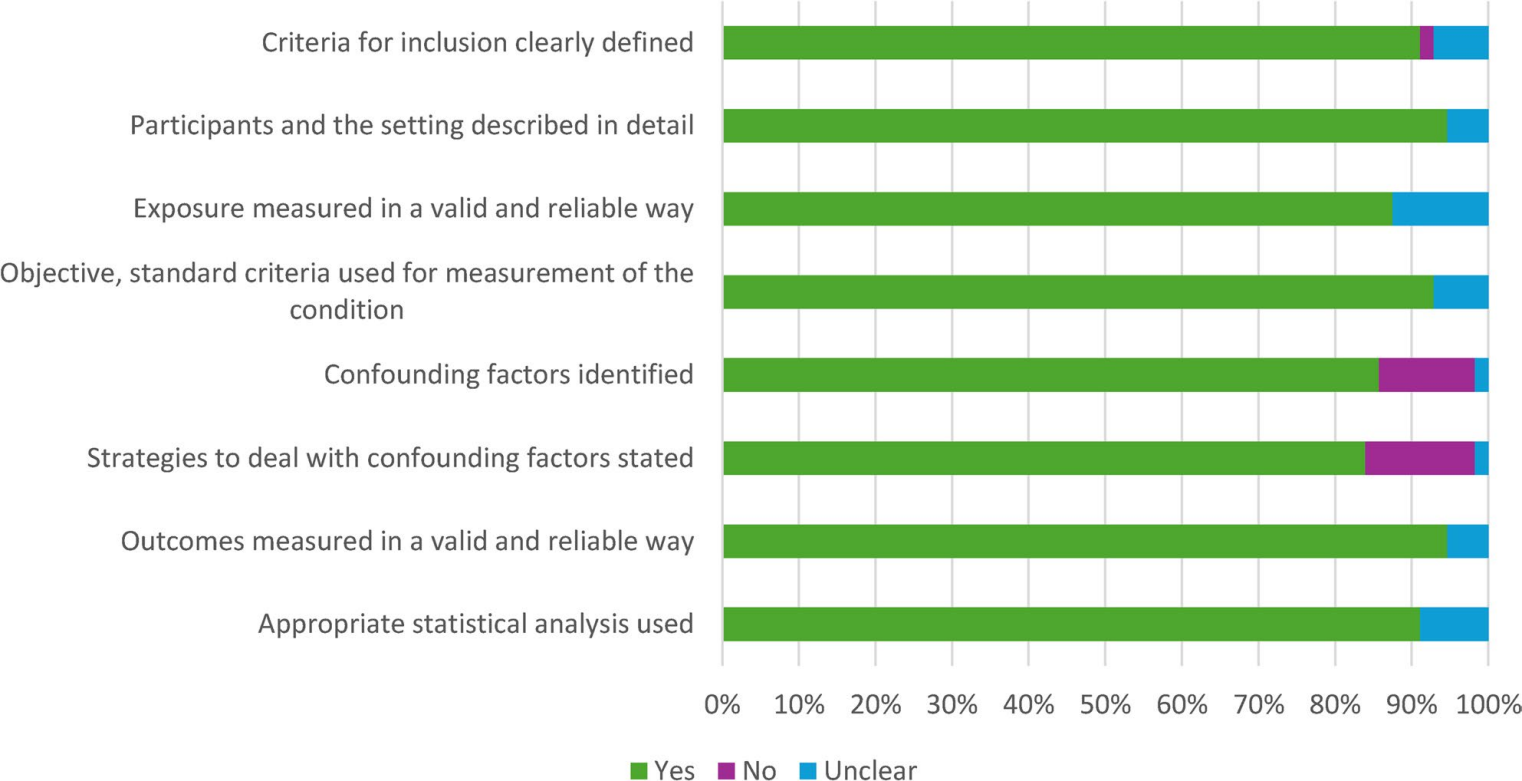
Summary of each item in the risk of bias tool for all included studies

Figure 5 summarises each item in the ROB tool for all included studies. The detailed evaluations for each study are presented in Supplementary File 5. Fifty-three (94.6%) of the included studies had a low risk of bias, three (5.4%) had a moderate risk, and none were classified as high risk. The average ROB score was 7.2, indicating a low risk.

## Discussion

We synthesised the evidence on the direction and magnitude of the association between health behaviours and life satisfaction among older adults. Regarding the direction of association, most of the included studies suggest that good sleep (quality sleep and/or 7–8 h of sleep), higher physical activity level, and fruit and vegetable intake were associated with higher life satisfaction, while a few studies reported that smoking and alcohol use were associated with lower life satisfaction. For the magnitude of association, only four cross-sectional bivariate studies on the association between physical activity and life satisfaction met the inclusion criteria for meta-analysis. The meta-analysis showed a weak but significant correlation between higher physical activity levels and higher life satisfaction.

The review was grounded on the United Nations’ SDG three of ensuring healthy lives and promoting well-being, especially among older adults [25]. From this perspective, we conceptualised that health behaviours are the foundation for enhancing the healthy ageing experience. Guided by the biopsychosocial framework [20], the findings were interpreted by highlighting how health behaviours can affect life satisfaction through biological and psychosocial lenses.

The narrative synthesis showed that the association between good sleep and higher life satisfaction was reported in 77.3% of unique analyses, making it the most significant among the reviewed health behaviours. Although in another outcome, similar reviews have reported that good sleep is independently associated with a lower risk of all-cause mortality and cardiovascular events [100] and with the quality of life [101]. An umbrella review reported that good sleep is associated with good health outcomes [102]. These findings, along with ours, highlight the critical importance of good sleep in the ageing population, as their biopsychosocial pathways are proximal to life satisfaction than many other health behaviours [103], whose benefits (e.g., physical activity) accumulate gradually over time. Good sleep is essential for the body’s restorative processes, especially among older adults, to reduce the rate of age-related decline [104]. Quality sleep was associated with better immune function, higher mental acuity/cognitive function, and a lower risk of chronic conditions, ultimately leading to higher life satisfaction [97, 99, 105]. Sleep also improves mental health functioning by mitigating disordered mood states, including anger, depression, and anxiety, thereby promoting social interaction and fostering greater life satisfaction [19, 106]. Additionally, it can improve social and psychological factors, such as self-image, boost self-esteem, and contribute to a positive outlook, which are essential for higher life satisfaction [22].

The physical activity was positively associated with life satisfaction in 69.1% of the unique analyses. However, the pooled effect size of the association is small (*r* = 0.12) and significant. Although the meta-analysis included only studies that assessed physical activity and life satisfaction and reported zero-order correlations, limitations inherent in measurement and study design likely attenuated the observed effect. First, the assessment of the constructs relied on self-report measures, which are prone to recall bias and social desirability, potentially diluting the pooled coefficient [107]. Additionally, the cross-sectional design of the included studies means that associations were captured at a single point in time [108], potentially underestimating the true relationship. The influence of physical activity on life satisfaction often unfolds gradually and may not be fully reflected in cross-sectional snapshots [85]. As a result, cross-sectional correlations tend to be smaller than associations observed in longitudinal or intervention designs. Finally, the use of zero-order correlations means that the pooled effect does not account for other determinants of life satisfaction, including demographic and socioeconomic factors, health status, and social support [11–13].

Notwithstanding the small effect size, it is established that physical activity is beneficial to health by maintaining bone and muscle strength, enhancing cardiovascular and brain health and reducing the risks of non-communicable diseases [109–111]. Maintaining physical activity is also important for older adults to experience greater life satisfaction through psychosocial gains [112]. Physical activity provides older adults with an environment to meet and make friends, creating a sense of belonging and social integration [23, 112]. It improves mood, self-image, and self-esteem and fosters a sense of control and self-efficacy [113, 114]. Moreover, physical activity provides an alternative form of engagement for retired older adults, helping them maintain their sense of purpose and meaning [24].

A healthy diet can enhance older adults’ life satisfaction by improving their health. This improvement can be achieved by supplying essential nutrients that support body function, maintain endocrine balance, boost energy levels and the immune system, and prevent chronic diseases [27, 115, 116]. Maintaining a healthy diet can enhance body image, boost self-esteem, and improve mood [117, 118].

Alcohol use and smoking show inconsistent associations with life satisfaction, with a few of the included studies identifying them as drivers of lower life satisfaction. The inconsistent associations may reflect cultural and social variations, as smoking or alcohol use carries different meanings and social consequences across countries [119, 120]. In societies where moderate alcohol use is embedded in social rituals and communal gatherings, drinking may support social engagement, while in some cultures, alcohol use is discouraged or stigmatised. Similarly, smoking may be viewed as a socially acceptable habit in some societies, while in others it may be prohibited. Additionally, variability in measurement approaches across studies likely contributes to inconsistent associations. Instruments differ in whether they assess habit versus frequency/ quantity, or social versus problematic use. For example, measures that distinguish moderate social drinking from hazardous or binge drinking are more likely to detect negative associations [65, 66], while those that capture only broad categories (e.g., “drinks alcohol: yes/no”) obscure meaningful differences in use patterns [9, 59].

Although these habits may serve as coping behaviours or social connections, they have severe health implications. Excessive alcohol use and smoking are known risk factors for various diseases, such as multiple organ cancers, diabetes, hypertension, dementia, and chronic lung, liver, and kidney diseases [120, 121]. Additionally, long-term drinking and smoking have been associated with mood disorders such as depression and anxiety [122, 123]. Harmful alcohol use can also lead to suicidal ideation, violence, and aggression, and when consumed before driving, it increases the risk of accidents [120]. Furthermore, smokers may face social isolation as others avoid them due to the smoke [124]. Avoiding these health and psychosocial effects of smoking and alcohol drinking could enhance life satisfaction among older adults. Even if these habits provide immediate gratification, the accumulation of their effects leads to chronic diseases with a remarkable reduction in life satisfaction [30, 65, 120, 121].

There is potential for bidirectionality, where life satisfaction functions as both an outcome and a determinant of behavioural patterns in later life. For instance, Lappan et al. [63] reported that being a smoker longitudinally predicted lower life satisfaction, and greater life satisfaction also longitudinally predicted a reduced likelihood of smoking. While bidirectionality appears practically true, health behaviours may first influence life satisfaction before the bidirectional cycle begins. Moreover, it appears practically impossible to develop a direct intervention to improve life satisfaction without modifying health behaviours and other determinants [9, 18].

### Strengths and limitations

This study is the first systematic review and meta-analysis to synthesise evidence on the association between a broad range of health behaviours and life satisfaction among community-dwelling older adults, addressing a clear gap in the literature. Another strength of this review lies in its comprehensive and transparent methodological rigour, including adherence to PRISMA guidelines, protocol registration, extensive searches across multiple major databases, and dual independent screening, data extraction, and risk of assessment with conflict resolution by a third reviewer. The comprehensiveness of the methodology enhances the reproducibility and credibility of the findings. Finally, the review is grounded in a biopsychosocial conceptual framework and anchored on the United Nations’ SDG three, offering a theoretically and practically informed interpretation of the association between life satisfaction and health behaviours.

The data in the included studies were collected using questionnaires, which may be prone to self-report errors, particularly recall bias. Moreover, questions on habits and health behaviours, such as smoking and alcoholism, are prone to social desirability biases. These biases may lead to either an underestimation or an overestimation of the effect size. No causal inferences can be made from the review results because we included only observational studies. Causality is typically drawn from randomised, controlled, interventional studies or trials. Study selection was limited to the English language and only observational studies, leading to the exclusion of other potential articles outside this scope.

The limited number of studies that assessed physical activity and life satisfaction with the same instruments restricted our ability to include more studies in the meta-analysis, potentially reducing statistical power and increasing uncertainty in the pooled associations. We were unable to complete meta-analyses on smoking, alcohol consumption, diet, and sleep due to the wide variability in the instruments used to measure both life satisfaction and the health behaviours. The reliance on narrative synthesis means that conclusions must be interpreted cautiously, as narrative approaches cannot account for between-study variability in the same systematic way that meta-analytic techniques allow, limiting comparability across studies.

### Implications of findings

The review’s outcomes have provided policymakers with valuable evidence to support healthy behaviours, such as physical activity, a healthy diet, and good sleep, to enhance the quality of life for older adults, aligning with the United Nations’ SDG three [25]. To promote healthy behaviours, it may be necessary to ensure access to nutritious food, create environments that facilitate physical activity, and campaign against risky behaviours.

Healthcare workers and caregivers can integrate the study findings into their evidence-based practices. These findings may inspire the incorporation of healthy behaviour practices into routine care tailored to the needs of older adults. As health behaviours are modifiable factors that individuals can control, adopting them may help older adults achieve higher life satisfaction.

Finally, the review has bridged the literature gap on the synthesis of the association between health behaviours and life satisfaction. However, future studies should employ a standardised instrument to assess health behaviours and life satisfaction, enabling cross-study comparisons and meta-synthesis of the research findings. Additionally, reporting both bivariate and multivariate analyses would provide a better understanding of the crude and adjusted effects of health behaviours on life satisfaction.

## Conclusions

Quality sleep and/or 7–8 h of sleep, a higher physical activity level, and regularly eating fruit and vegetables and/or a balanced diet are significantly associated with higher life satisfaction. In contrast, both cross-sectional and longitudinal studies sparsely supported the influence of smoking and alcohol consumption on lower life satisfaction. The review provides evidence for policymakers, healthcare workers, caregivers, and society to encourage healthy behaviours and foster healthy ageing. Future studies should use standardised instruments to assess health behaviours and life satisfaction.

## Supplementary Information

Supplementary Material 1.

## Data Availability

The data supporting this study’s findings can be found in Supplementary Files 3 and 4.

## Abbreviations

APA: American Psychological Association
CINAHL: Cumulative Index to Nursing and Allied Health Literature
CMA: Comprehensive Meta-Analysis
JBI: Joanna Briggs Institute
MEDLINE: Medical Literature Analysis and Retrieval System Online
MeSH: Medical Subject Headings
MOOSE: Meta-Analyses and Systematic Reviews of Observational Studies
PRISMA: Preferred Reporting Items for Systematic Review and Meta-Analysis
PROSPERO: International Prospective Register of Systematic Reviews
ROB: Risk of Bias Assessment
SDG: Sustainable Development Goal
Tau^2^: Tau-squared
