# Supplementary material for: Association between life satisfaction and health behaviours among older adults: a systematic review and meta-analysis
Source: Int J Behav Nutr Phys Act. 2026 Jan 26;23:15. doi: 10.1186/s12966-026-01877-1 (PMC12924400; doi:10.1186/s12966-026-01877-1)
Supplement: Supplementary file 1 — Supplementary Material 1. [file 12966_2026_1877_MOESM1_ESM.docx]

**Supplementary File 1:** PRISMA 2020 checklist, Page et al. (2021).

| **Section and Topic** | **Item #** | **Checklist item** | **Page** |
| --- | --- | --- | --- |
| **TITLE** | | |  |
| Title | 1 | Identify the report as a systematic review. | 1 |
| **ABSTRACT** | | |  |
| Abstract | 2 | See the PRISMA 2020 for Abstracts checklist. | 2 |
| **INTRODUCTION** | | |  |
| Rationale | 3 | Describe the rationale for the review in the context of existing knowledge. | 3-5 |
| Objectives | 4 | Provide an explicit statement of the objective(s) or question(s) the review addresses. | 4-5 |
| **METHODS** | | |  |
| Eligibility criteria | 5 | Specify the inclusion and exclusion criteria for the review and how studies were grouped for the syntheses. | 6 |
| Information sources | 6 | Specify all databases, registers, websites, organisations, reference lists and other sources searched or consulted to identify studies. Specify the date when each source was last searched or consulted. | 6 |
| Search strategy | 7 | Present the full search strategies for all databases, registers and websites, including any filters and limits used. | 6-7 & Suppl 2 |
| Selection process | 8 | Specify the methods used to decide whether a study met the inclusion criteria of the review, including how many reviewers screened each record and each report retrieved, whether they worked independently, and if applicable, details of automation tools used in the process. | 7 |
| Data collection process | 9 | Specify the methods used to collect data from reports, including how many reviewers collected data from each report, whether they worked independently, any processes for obtaining or confirming data from study investigators, and if applicable, details of automation tools used in the process. | 7 |
| Data items | 10a | List and define all outcomes for which data were sought. Specify whether all results that were compatible with each outcome domain in each study were sought (e.g. for all measures, time points, analyses), and if not, the methods used to decide which results to collect. | 8 |
|  | 10b | List and define all other variables for which data were sought (e.g. participant and intervention characteristics, funding sources). Describe any assumptions made about any missing or unclear information. | 8 |
| Study risk of bias assessment | 11 | Specify the methods used to assess risk of bias in the included studies, including details of the tool(s) used, how many reviewers assessed each study and whether they worked independently, and if applicable, details of automation tools used in the process. | 8 |
| Effect measures | 12 | Specify for each outcome the effect measure(s) (e.g. risk ratio, mean difference) used in the synthesis or presentation of results. | 8-9 |
| Synthesis methods | 13a | Describe the processes used to decide which studies were eligible for each synthesis (e.g. tabulating the study intervention characteristics and comparing against the planned groups for each synthesis (item #5)). | 8-9 |
|  | 13b | Describe any methods required to prepare the data for presentation or synthesis, such as handling of missing summary statistics, or data conversions. | 8-9 |
|  | 13c | Describe any methods used to tabulate or visually display results of individual studies and syntheses. | 8-9 |
|  | 13d | Describe any methods used to synthesise results and provide a rationale for the choice(s). If meta-analysis was performed, describe the model(s), method(s) to identify the presence and extent of statistical heterogeneity, and software package(s) used. | 8-9 |
|  | 13e | Describe any methods used to explore possible causes of heterogeneity among study results (e.g. subgroup analysis, meta-regression). | 10 |
|  | 13f | Describe any sensitivity analyses conducted to assess robustness of the synthesised results. | 10 |
| Reporting bias assessment | 14 | Describe any methods used to assess risk of bias due to missing results in a synthesis (arising from reporting biases). | 8 |
| Certainty assessment | 15 | Describe any methods used to assess certainty (or confidence) in the body of evidence for an outcome. | NA |
| **RESULTS** | | |  |
| Study selection | 16a | Describe the results of the search and selection process, from the number of records identified in the search to the number of studies included in the review, ideally using a flow diagram. | 10 |
|  | 16b | Cite studies that might appear to meet the inclusion criteria, but which were excluded, and explain why they were excluded. | 10 |
| Study characteristics | 17 | Cite each included study and present its characteristics. | 10-11 |
| Risk of bias in studies | 18 | Present assessments of risk of bias for each included study. | 17 |
| Results of individual studies | 19 | For all outcomes, present, for each study: (a) summary statistics for each group (where appropriate) and (b) an effect estimate and its precision (e.g. confidence/credible interval), ideally using structured tables or plots. | 11-17 |
| Results of syntheses | 20a | For each synthesis, briefly summarise the characteristics and risk of bias among contributing studies. | 11-17 |
|  | 20b | Present results of all statistical syntheses conducted. If meta-analysis was done, present for each the summary estimate and its precision (e.g. confidence/credible interval) and measures of statistical heterogeneity. If comparing groups, describe the direction of the effect. | 11-17 |
|  | 20c | Present results of all investigations of possible causes of heterogeneity among study results. | 11-17 |
|  | 20d | Present results of all sensitivity analyses conducted to assess the robustness of the synthesised results. | 11-17 |
| Reporting biases | 21 | Present assessments of risk of bias due to missing results (arising from reporting biases) for each synthesis assessed. | 11-17 |
| Certainty of evidence | 22 | Present assessments of certainty (or confidence) in the body of evidence for each outcome assessed. | NA |
| **DISCUSSION** | | |  |
| Discussion | 23a | Provide a general interpretation of the results in the context of other evidence. | 17-21 |
|  | 23b | Discuss any limitations of the evidence included in the review. | 22 |
|  | 23c | Discuss any limitations of the review processes used. | 22 |
|  | 23d | Discuss implications of the results for practice, policy, and future research. | 22-23 |
| **OTHER INFORMATION** | | |  |
| Registration and protocol | 24a | Provide registration information for the review, including register name and registration number, or state that the review was not registered. | 5 |
|  | 24b | Indicate where the review protocol can be accessed, or state that a protocol was not prepared. | 5 |
|  | 24c | Describe and explain any amendments to information provided at registration or in the protocol. | 5 |
| Support | 25 | Describe sources of financial or non-financial support for the review, and the role of the funders or sponsors in the review. | 25 |
| Competing interests | 26 | Declare any competing interests of review authors. | 24 |
| Availability of data, code and other materials | 27 | Report which of the following are publicly available and where they can be found: template data collection forms; data extracted from included studies; data used for all analyses; analytic code; any other materials used in the review. | 24 |

**Supplementary File 2:** Search Strategies for the five databases

|  | **MEDLINE Database search strategy** |  |
| --- | --- | --- |
| **ID#** | **Search terms** | **Results** |
| 1 | exp Aged/ [MeSH] | 3,620,694 |
| 2 | exp Geriatrics/ [MeSH] | 32,057 |
| 3 | ((old* or age*) adj3 (people* or person* or adult* or women* or men* or citizen* or residen*)).mp. | 581,020 |
| 4 | (aged or elderly or senior* or geriatric*).mp. | 622,5234 |
| 5 | or/1-4 | 6,332,860 |
| 6 | exp personal satisfaction/ [MeSH] | 26,752 |
| 7 | ((life or lives or personal) adj2 satisf*).mp. | 35,195 |
| 8 | or/6-7 | 35,737 |
| 9 | exp Health Behavior/ [MeSH] | 377,325 |
| 10 | exp Healthy Lifestyle/ [MeSH] | 13,862 |
| 11 | (health* adj3 (behavior* or behaviour* or lifestyle*)).mp. | 130,586 |
| 12 | exp Diet/ [MeSH] | 348,121 |
| 13 | (diet* or nutrition or fruit* or vegetable*).mp. | 1,144,574 |
| 14 | (food* adj3 (choice* or consum* or pattern* or intak*)).mp. | 100,459 |
| 15 | exp Sleep/ [MeSH] | 105,400 |
| 16 | sleep*.mp. | 247,264 |
| 17 | exp Smoking/ [MeSH] | 165,517 |
| 18 | exp "Tobacco Use"/ [MeSH] | 10,882 |
| 19 | (smok* or tobacco).mp. | 410,635 |
| 20 | exp Alcohol Drinking/ | 81,137 |
| 21 | exp Alcohol-Related Disorders/ [MeSH] | 124,515 |
| 22 | (alcohol* adj2 (drink* or intoxicat* or use* or abus* or misus* or risk* or consum*)).mp. | 167,149 |
| 23 | (drink* adj2 (excess or heavy or heavily or harm or harmful or hazard* or binge or harmful or problem*)).mp. | 22,244 |
| 24 | (alcoholic* or alcoholism).mp. | 177,086 |
| 25 | exp Exercise/ [MeSH] | 265,820 |
| 26 | Physical Exertion/ [MeSH] | 57,800 |
| 27 | exp Physical Fitness/ [MeSH] | 38,404 |
| 28 | exp Sports/ [MeSH] | 229,018 |
| 29 | (exercis* or fitness or sport* or walk*).mp. | 709,430 |
| 30 | (physical* adj2 activ*).mp. | 150,647 |
| 31 | or/9-30 | 3,063,247 |
| 32 | comparative study/ [MeSH] | 1,936,453 |
| 33 | Follow-Up Studies/ [MeSH] | 706,980 |
| 34 | Time Factors/ [MeSH] | 1,244,208 |
| 35 | chang*.mp. | 3,430,541 |
| 36 | evaluat*.mp. | 4,420,456 |
| 37 | reviewed.mp. | 581,882 |
| 38 | prospective*.mp. | 1,013,390 |
| 39 | retrospective*.mp. | 1,396,011 |
| 40 | baseline.mp. | 686,430 |
| 41 | cohort.mp. | 887,149 |
| 42 | case series.mp. | 90,597 |
| 43 | or/32-42 | 11,131,236 |
| 44 | 5 and 8 and 31 and 43 | 2,727 |
| 45 | limit 44 to English language | 2,597 |

| **Web of Science Database Search Strategy** | | |
| --- | --- | --- |
| **ID#** | **Search terms** | **Results** |
| 26 | #3 AND #4 AND #15 AND #24 and English (Languages) | 1,379 |
| 25 | #3 AND #4 AND #15 AND #24 | 1,461 |
| 24 | #16 OR #17 OR #18 OR #19 OR #20 OR #21 OR #22 OR #23 | 20,252,128 |
| 23 | TS=(case series) | 386,085 |
| 22 | TS=(cohort) | 1,151,769 |
| 21 | TS=(baseline) | 985,509 |
| 20 | TS=(retrospective*) | 1,182,978 |
| 19 | TS=(prospective*) | 1,055,878 |
| 18 | TS=(reviewed) | 4,154,617 |
| 17 | TS=(evaluat*) | 8,471,794 |
| 16 | TS=(chang*) | 7,938,274 |
| 15 | #5 OR #6 OR #7 OR #8 OR #9 OR #10 OR #11 OR #12 OR #13 OR #14 | 4,620,910 |
| 14 | TS=(physical* NEAR/2 activ*) | 314,910 |
| 13 | TS=(exercis* or fitness or sport* or walk*) | 1,370,999 |
| 12 | TS=(alcoholic* or alcoholism) | 142,766 |
| 11 | TS=(drink* NEAR/2 (excess or heavy or heavily or harm or harmful or hazard* or binge or harmful or problem*)) | 35,595 |
| 10 | TS=(alcohol* NEAR/2 (drink* or intoxicat* or use* or abus* or misus* or risk* or consum*)) | 202,309 |
| 9 | TS=(smok* or tobacco) | 583,551 |
| 8 | TS=(sleep*) | 382,056 |
| 7 | TS=(food* NEAR/3 (choice* or consum* or pattern* or intak*)) | 200,409 |
| 6 | TS=(diet* or nutrition or fruit* or vegetable*) | 1,961,484 |
| 5 | TS=(health* NEAR/3 (behavior or behaviour* or lifestyle*)) | 146,295 |
| 4 | TS=((life or lives or personal) NEAR/2 satisf*) | 45,401 |
| 3 | #2 OR #1 | 5,331,405 |
| 2 | TS=(aged or elderly or senior* or geriatric*) | 5,158,037 |
| 1 | TS=((old* or age*) NEAR/3 (people* or person* or adult* or women* or men* or citizen* or resid*) | 913,150 |

|  | **CINAHL Database Search Strategy** | |  |
| --- | --- | --- | --- |
| **ID#** | **Search terms** | **Search options** | **Results** |
| S44 | S5 AND S8 AND S30 AND S42 | **Expanders** - Apply equivalent subjects  **Narrow by Language**: - English  **Search modes** - Proximity | 1,237 |
| S43 | S5 AND S8 AND S30 AND S42 | **Expanders** - Apply equivalent subjects  **Search modes** - Proximity | 1,295 |
| S42 | S31 OR S32 OR S33 OR S34 OR S35 OR S36 OR S37 OR S38 OR S39 OR S40 OR S41 | **Expanders** - Apply equivalent subjects  **Search modes** - Proximity | 2,983,370 |
| S41 | case series | **Expanders** - Apply equivalent subjects  **Search modes** - Proximity | 38,156 |
| S40 | cohort | **Expanders** - Apply equivalent subjects  **Search modes** - Proximity | 323,285 |
| S39 | baseline | **Expanders** - Apply equivalent subjects  **Search modes** - Proximity | 238,816 |
| S38 | retrospective* | **Expanders** - Apply equivalent subjects  **Search modes** - Proximity | 438,697 |
| S37 | prospective* | **Expanders** - Apply equivalent subjects  **Search modes** - Proximity | 650,349 |
| S36 | reviewed | **Expanders** - Apply equivalent subjects  **Search modes** - Proximity | 152,173 |
| S35 | evaluat* | **Expanders** - Apply equivalent subjects  **Search modes** - Proximity | 1,710,702 |
| S34 | chang* | **Expanders** - Apply equivalent subjects  **Search modes** - Proximity | 727,618 |
| S33 | (MH "Time Factors+") | **Expanders** - Apply equivalent subjects  **Search modes** - Proximity | 188,930 |
| S32 | (MH "Prospective Studies+") | **Expanders** - Apply equivalent subjects  **Search modes** - Proximity | 541,587 |
| S31 | (MH "Comparative Studies+") | **Expanders** - Apply equivalent subjects  **Search modes** - Proximity | 495,474 |
| S30 | S9 OR S10 OR S11 OR S12 OR S13 OR S14 OR S15 OR S16 OR S17 OR S18 OR S19 OR S20 OR S21 OR S22 OR S23 OR S24 OR S25 OR S26 OR S27 OR S28 OR S29 | **Expanders** - Apply equivalent subjects  **Search modes** - Proximity | 1,195,023 |
| S29 | physical* N2 activ* | **Expanders** - Apply equivalent subjects  **Search modes** - Proximity | 108,707 |
| S28 | exercis* of fitness or sport* or walk* | **Expanders** - Apply equivalent subjects  **Search modes** - Proximity | 357,512 |
| S27 | (MH "Sports+") | **Expanders** - Apply equivalent subjects  **Search modes** - Proximity | 95,134 |
| S26 | (MH "Physical Fitness+") | **Expanders** - Apply equivalent subjects  **Search modes** - Proximity | 22,712 |
| S25 | (MH "Physical Activity+") | **Expanders** - Apply equivalent subjects  **Search modes** - Proximity | 56,969 |
| S24 | (MH "Exertion+") | **Expanders** - Apply equivalent subjects  **Search modes** - Proximity | 111,768 |
| S23 | (MH "Exercise+") | **Expanders** - Apply equivalent subjects  **Search modes** - Proximity | 135,963 |
| S22 | alcoholic* or alcoholism | **Expanders** - Apply equivalent subjects  **Search modes** - Proximity | 47,644 |
| S21 | drink* N2 (excess or heavy or heavily or harm or harmful or hazard* or binge or harmful or problem* | **Expanders** - Apply equivalent subjects  **Search modes** - Proximity | 14,120 |
| S20 | alcohol* N2 (drink* or intoxicat* or use* or abus* or misus* or risk* or consum*) | **Expanders** - Apply equivalent subjects  **Search modes** - Proximity | 86,883 |
| S19 | (MH "Alcohol-Related Disorders+") | **Expanders** - Apply equivalent subjects  **Search modes** - Proximity | 44,150 |
| S18 | (MH "Alcohol Drinking+") | **Expanders** - Apply equivalent subjects  **Search modes** - Proximity | 37,976 |
| S17 | smok* or tobacco | **Expanders** - Apply equivalent subjects  **Search modes** - Proximity | 155,059 |
| S16 | (MH "Smoking+") | **Expanders** - Apply equivalent subjects  **Search modes** - Proximity | 68,918 |
| S15 | sleep* | **Expanders** - Apply equivalent subjects  **Search modes** - Proximity | 98,314 |
| S14 | (MH "Sleep+") | **Expanders** - Apply equivalent subjects  **Search modes** - Proximity | 36,191 |
| S13 | food* N3 (choice* or consum* or pattern* or intak*) | **Expanders** - Apply equivalent subjects  **Search modes** - Proximity | 50,300 |
| S12 | diet or nutrition or fruit* or vegetable* | **Expanders** - Apply equivalent subjects  **Search modes** - Proximity | 378,602 |
| S11 | (MH "Diet+") | **Expanders** - Apply equivalent subjects  **Search modes** - Proximity | 147,602 |
| S10 | health* N3 (behavior* or behaviour* or lifestyle*) | **Expanders** - Apply equivalent subjects  **Search modes** - Proximity | 109,795 |
| S9 | (MH "Health Behavior+") | **Expanders** - Apply equivalent subjects  **Search modes** - Proximity | 149,438 |
| S8 | S6 OR S7 | **Expanders** - Apply equivalent subjects  **Search modes** - Proximity | 51,671 |
| S7 | (life or lives or personal) N2 satisf* | **Expanders** - Apply equivalent subjects  **Search modes** - Proximity | 24,170 |
| S6 | (MH "Personal Satisfaction+") | **Expanders** - Apply equivalent subjects  **Search modes** - Proximity | 46,011 |
| S5 | S1 OR S2 OR S3 OR S4 | **Expanders** - Apply equivalent subjects  **Search modes** - Proximity | 1,298,022 |
| S4 | aged or elderly or senior or geriatric | **Expanders** - Apply equivalent subjects  **Search modes** - Proximity | 1,202,133 |
| S3 | (old* or age*) N3 (people* or person* or adult* or women* or men* or citizen* or residen*) | **Expanders** - Apply equivalent subjects  **Search modes** - Proximity | 332,898 |
| S2 | (MH "Geriatrics+") | **Expanders** - Apply equivalent subjects  **Search modes** - Proximity | 7,102 |
| S1 | (MH "Aged+") | **Expanders** - Apply equivalent subjects  **Search modes** - Proximity | 978,208 |

|  | **APA PsycINFO Database Search Strategy** | |
| --- | --- | --- |
| **ID#** | **Search term** | **Results** |
| 1 | exp Aging/ or exp Older Adulthood/ | 112,709 |
| 2 | exp Geriatrics/ | 16,023 |
| 3 | ((old* or age*) adj3 (people* or person* or adult* or women* or men* or citizen* or resident*)).mp. | 249,336 |
| 4 | (aged or elderly or senior* or geriatric*).mp. | 744,627 |
| 5 | or/1-4 | 871,627 |
| 6 | exp Life Satisfaction/ | 15,866 |
| 7 | ((life or lives or personal) adj2 satisf*).mp. | 38,386 |
| 8 | or/6-7 | 40,152 |
| 9 | exp Health Behavior/ | 46,667 |
| 10 | (health* adj3 (behavior* or behaviour* or lifestyle*)).mp. | 98,998 |
| 11 | exp Diets/ | 21,796 |
| 12 | (diet* or nutrition or fruit* or vegetable*).mp. | 101,480 |
| 13 | (food* adj3 (choice* or consum* or pattern* or intak*)).mp. | 32,695 |
| 14 | exp Sleep/ | 47,807 |
| 15 | sleep*.mp. | 109,163 |
| 16 | exp tobacco smoking/ | 38,767 |
| 17 | (smok* or tobacco).mp. | 84,115 |
| 18 | exp "Alcohol Use"/ | 32,877 |
| 19 | exp "alcohol use disorder"/ | 59,792 |
| 20 | (alcohol* adj2 (drink* or intoxicat* or use* or abus* or misus* or risk* or consum*)).mp. | 107,721 |
| 21 | (drink* adj2 (excess or heavy or heavily or harm or harmful or hazard* or binge or harmful or problem*)).mp. | 20,332 |
| 22 | (alcoholic* or alcoholism).mp. | 67,317 |
| 23 | exp physical activity/ | 58,178 |
| 24 | exp sports/ | 47,982 |
| 25 | (exercis* or fitness or sport* or walk*).mp. | 194,064 |
| 26 | (physical* adj2 activ*).mp. | 58,081 |
| 27 | or/9-26 | 687,811 |
| 28 | exp Followup Studies/ | 12,406 |
| 29 | chang*.mp. | 853,853 |
| 30 | evaluat*.mp. | 743,581 |
| 31 | reviewed.mp. | 102,892 |
| 32 | prospective.mp. | 99,774 |
| 33 | retrospective*.mp. | 73,266 |
| 34 | baseline.mp. | 153,198 |
| 35 | cohort.mp. | 108,594 |
| 36 | case series.mp. | 5,463 |
| 37 | or/28-36 | 1,752,148 |
| 38 | 5 and 8 and 27 and 37 | 1,010 |
| 39 | limit 38 to English language | 965 |

|  | **Global Health Database Search Strategy** | |  |
| --- | --- | --- | --- |
| **ID#** | **Search terms** | **Search options** | **Results** |
| S37 | S5 AND S6 AND S26 AND S35 | **Expanders** - Apply equivalent subjects  **Narrow by Language**: - English  **Search modes** - Proximity | 225 |
| S36 | S5 AND S6 AND S26 AND S35 | **Expanders** - Apply equivalent subjects  **Search modes** - Proximity | 248 |
| S35 | S27 OR S28 OR S29 OR S30 OR S31 OR S32 OR S33 OR S34 | **Expanders** - Apply equivalent subjects  **Search modes** - Proximity | 1,813,607 |
| S34 | case series | **Expanders** - Apply equivalent subjects  **Search modes** - Proximity | 12,745 |
| S33 | cohort | **Expanders** - Apply equivalent subjects  **Search modes** - Proximity | 206,818 |
| S32 | baseline | **Expanders** - Apply equivalent subjects  **Search modes** - Proximity | 138,355 |
| S31 | retrospective* | **Expanders** - Apply equivalent subjects  **Search modes** - Proximity | 149,488 |
| S30 | prospective* | **Expanders** - Apply equivalent subjects  **Search modes** - Proximity | 145,319 |
| S29 | reviewed | **Expanders** - Apply equivalent subjects  **Search modes** - Proximity | 107,380 |
| S28 | evaluat* | **Expanders** - Apply equivalent subjects  **Search modes** - Proximity | 923,723 |
| S27 | chang* | **Expanders** - Apply equivalent subjects  **Search modes** - Proximity | 673,112 |
| S26 | S7 OR S8 OR S9 OR S10 OR S11 OR S12 OR S13 OR S14 OR S15 OR S16 OR S17 OR S18 OR S19 OR S20 OR S21 OR S22 OR S23 OR S24 OR S25 | **Expanders** - Apply equivalent subjects  **Search modes** - Proximity | 1,547,496 |
| S25 | physical* N2 activ* | **Expanders** - Apply equivalent subjects  **Search modes** - Proximity | 87,603 |
| S24 | exercis* or fitness or sport* or walk* | **Expanders** - Apply equivalent subjects  **Search modes** - Proximity | 156,121 |
| S23 | DE "physical activity" | **Expanders** - Apply equivalent subjects  **Search modes** - Proximity | 68,685 |
| S22 | DE "physical fitness" | **Expanders** - Apply equivalent subjects  **Search modes** - Proximity | 7,974 |
| S21 | DE "exercise" | **Expanders** - Apply equivalent subjects  **Search modes** - Proximity | 41,972 |
| S20 | alcoholic* or alcoholism | **Expanders** - Apply equivalent subjects  **Search modes** - Proximity | 60,490 |
| S19 | drink* N2 (excess or heavy or heavily or harm or harmful or hazard* or binge or harmful or problem*) | **Expanders** - Apply equivalent subjects  **Search modes** - Proximity | 9,462 |
| S18 | alcohol* N2 (drink* or intoxicat* or use* or abus* or misus* or risk* or consum*) | **Expanders** - Apply equivalent subjects  **Search modes** - Proximity | 60,259 |
| S17 | DE "alcoholism" | **Expanders** - Apply equivalent subjects  **Search modes** - Proximity | 11,050 |
| S16 | DE "alcohol intake" | **Expanders** - Apply equivalent subjects  **Search modes** - Proximity | 39,785 |
| S15 | smok* or tobacco | **Expanders** - Apply equivalent subjects  **Search modes** - Proximity | 123,843 |
| S14 | DE "smoking" | **Expanders** - Apply equivalent subjects  **Search modes** - Proximity | 2,033 |
| S13 | sleep* | **Expanders** - Apply equivalent subjects  **Search modes** - Proximity | 33,763 |
| S12 | DE "sleep" | **Expanders** - Apply equivalent subjects  **Search modes** - Proximity | 15,248 |
| S11 | food* N3 (choice* or consum* or pattern* or intak*) | **Expanders** - Apply equivalent subjects  **Search modes** - Proximity | 142,374 |
| S10 | diet* or nutrition or fruit* or vegetable* | **Expanders** - Apply equivalent subjects  **Search modes** - Proximity | 1,268,009 |
| S9 | DE "diet" | **Expanders** - Apply equivalent subjects  **Search modes** - Proximity | 66,870 |
| S8 | health* N3 (behavior* or behaviour* or lifestyle*) | **Expanders** - Apply equivalent subjects  **Search modes** - Proximity | 69,469 |
| S7 | DE "health behaviour" | **Expanders** - Apply equivalent subjects  **Search modes** - Proximity | 22,070 |
| S6 | (life or lives or personal) N2 satisf* | **Expanders** - Apply equivalent subjects  **Search modes** - Proximity | 3,255 |
| S5 | S1 OR S2 OR S3 OR S4 | **Expanders** - Apply equivalent subjects  **Search modes** - Proximity | 408,302 |
| S4 | aged or elderly or senior* or geriatric* | **Expanders** - Apply equivalent subjects  **Search modes** - Proximity | 328,692 |
| S3 | (old* or age*) N3 (people* or person* or adult* or women* or men* or citizen* or resident*) | **Expanders** - Apply equivalent subjects  **Search modes** - Proximity | 238,990 |
| S2 | DE "geriatrics" | **Expanders** - Apply equivalent subjects  **Search modes** - Proximity | 1,853 |
| S1 | DE "elderly" OR DE "elderly patients" OR DE "frail elderly" | **Expanders** - Apply equivalent subjects  **Search modes** - Proximity | 82,331 |

**Supplementary File 3:** Characteristics of the included studies (n = 56)

| **First author last name, Year, &**  **Country** | **Title** | **Dataset name** | **Sample size** | **Age range, [mean] years** | **%**  **Female** | **Smoking operationalisation/ instrument** | **Alcohol drinking operationalisation/ instrument** | **Physical activity operationalisation/ instrument** | **Diet/Nutrition operationalisation/ instrument** | **Sleep operationalisation/ instrument** | **Life satisfaction operationalisation/ instrument** |
| --- | --- | --- | --- | --- | --- | --- | --- | --- | --- | --- | --- |
| *Achour, 2011  France | Level of physical activity at the age of 65 predicts successful aging seven years later: the PROOF study | PROOF | 686 | ≥65  [72.9] | 59.5 |  |  | Activity index calculated from the Population Physical Activity Questionnaire (POPAQ) |  |  | Single-item question: "Are you generally satisfied with your life" rated on a zero to 10 Visual Analogue Scale |
| An, 2020  Taiwan | The relationships between physical activity and life satisfaction and happiness among young, middle-aged, and older adults | NA | 643 | [73.7] | 60.3 |  |  | International Physical Activity Questionnaire (IPAQ) short form and categorised into low-active, moderate-active, and high-active. |  |  | Single-item question whose response was rated on an 11-point scale from 0 to 10: “In general, how satisfied are you with your life?” |
| Andre, 2017  Norway | Is there an association between food patterns and life satisfaction among Norway's inhabitants ages 65 years and older? | HUNT | 11619 | ≥65 | 54.2 |  |  |  | Unhealthy food cluster and Healthy food cluster/ A single question about how often they normally eat various foods and drink liquids |  | Single-item question: "Thinking about your life at the moment, would you say that you by and large are satisfied with life, or are you mostly dissatisfied? on a 7-point Likert Scale with 1 = very satisfied and 7 = very dissatisfied |
| Bae, 2017  United States of America | Physical activity levels and well-being in older adults | MIDUS | 1176 | 60–84  [70.8] | 52.2 |  |  | How often do you engage in vigorous, moderate, and light physical activities? Each intensity was rated on a 6-point Likert-type scale, and the items ranged from never (1) to several times a week (6). |  |  | Single-item question: “Using a scale from 0 to 10, where 0 means the worst possible life overall and 10 means the best possible life overall, how would you rate your life overall these days?” |
| Banjare, 2015  India | Factors associated with the life satisfaction amongst the rural elderly in Odisha, India | NA | 310 | ≥60  [69.3] | 50.6 | Do you smoke? Yes or no | Do you consume alcohol? Yes or no |  |  |  | Single-item question “Taking all things together, how would you say you are these days”? The responses were 1. very happy, 2. happy, 3. neither happy nor unhappy, 4. unhappy and 5. very unhappy. |
| Bertelli-Costa, 2021  Brazil | Life satisfaction and participation  among community-dwelling older  adults: Data from the FIBRA study | FIBRA | 2344 | ≥65  [72.3] | 65.6 |  |  | Global, Leisure-time, Domestic, Occupational, and Transportation PA were rated on 3 levels: inactive, insufficiently active, and active using the Brazilian version of the Minnesota Leisure-Time Physical Activity Questionnaire |  |  | Single-item question “Are you satisfied with your life today?” with three response options: “little,” “somewhat,” or “very” |
| Bourque, 2005  Canada | Contextual effects on life satisfaction of older men and women | ACS | 958 | 65–94  [73.0] | 57 |  |  | Three-item question with a composite higher score equals higher PA. Q1: For a person your age, what is your level of physical activity? Light, moderate, or strenuous. Q2: To what extent is physical exercise important in the prevention of illness in older adults? Unimportant, somewhat important, moderately important, or very important. Q3: The number of times the participants exercised per week. |  |  | Five items pertaining to satisfaction with health, home, community, income, and life in general were measured on a 5-point scale ranging from not satisfied at all to very satisfied. |
| Bourque, 2007  Canada | Self-reported sensory impairment and life satisfaction in older French-speaking adults | ACS | 826 | 65–94  [74.0] | 64 |  |  | Three-item question with a composite higher score equals higher PA. Q1: For a person your age, would you say that you engage in: very little activity (1), enough physical activity (2), or much physical activity (3). Q2: How important is physical activity in preventing illness in older adults? Unimportant (1), somewhat important (2), quite important (3), very important (4). Q3: What is your level of physical activity? Light, moderate, or strenuous |  |  | Single-item question "Are you satisfied with your life in general?" on a 5-point scale ranging from not satisfied at all to very satisfied. |
| *Caligiuri, 2012  Canada | Changes in food group consumption and associations with self-rated diet, health, life satisfaction, and mental and physical functioning over 5 years in very old Canadian men: The Manitoba Follow-Up Study | MFUS | 736 | [79.4] |  |  |  |  | How often do you NOW eat/drink the four food groups: fruit & vegetable, grain product, milk & alternatives, and meat & alternatives? every day, most days, and rarely. Food group consumption change was defined as: Still every day, improved, still most days, and still rarely/decline. Grouped into remain daily/improved or poor/declined for inferential analysis |  | Single-item question “How would you describe your satisfaction with life in general at present?” with options: excellent, good, fair, poor, or bad. Life satisfaction change was grouped into change for the better or change for the worse for inferential analysis. |
| Cho, 2023  United States of America | Older adults’ advance aging and life satisfaction levels: effects of lifestyles and health capabilities | MIDUS | 290 | 60–78  [67.3] | 41.4 | Have you now or in the past smoked regularly? Yes or no | In the past month, have you had at least one drink of any alcoholic beverages such as beer, wine, wine coolers, or liquor? Yes or no | Do you engage in regular exercise or activity of any type for 20 minutes or more at least three times per week? Yes or no | Do you follow a special diet? Yes or no |  | Satisfaction With Life Scale |
| Colsher, 1990  United States of America | Elderly men with histories of heavy drinking: correlates and consequence | RHS | 1150 | ≥65  [73.4] |  |  | Never drinkers (denied ever having consumed alcohol), former drinkers (no alcohol consumption during the past year) or current drinkers (consumed alcohol in past year) |  |  |  | Not described |
| Crawford-Achour, 2014  France | Can subjective sleep quality, evaluated at the age of 73, have an influence on successful aging? The PROOF study | PROOF | 370 | 73  [73.2] | 53.8 |  |  |  |  | French version of the Pittsburgh Sleep Quality Index (PSQI). A high score indicates high sleep difficulty | Single-item question: "Are you generally satisfied with your current life" rated on a zero to 10 Visual Analogue Scale |
| †Di Gessa, 2023  United Kingdom | Health behaviors and mental health during the covid-19 pandemic: evidence from the English Longitudinal Study of Aging | ELSA | 4989 | ≥50  [70.8] | 57.1 |  | Respondents were asked in June/ July 2020 (baseline) if they had been drinking alcohol “less than usual,” “about the same,” or “more than usual” since the COVID-19 outbreak began in February 2020. | Respondents were asked in June/ July 2020 (baseline) if they had been doing physical activity “less than usual,” “about the same,” or “more than usual” since the COVID-19 outbreak began in February 2020. |  | Respondents were asked in June/ July 2020 (baseline) if they had been sleeping “less than usual,” “about the same,” or “more than usual” since the COVID-19 outbreak began in February 2020. | Office for National Statistics (ONS) wellbeing scale (“On a scale of 0 - 10, where 0 is ‘not at all’ and 10 is ‘very’, how satisfied are you with your life nowadays?”) |
| Fichten, 2004  Canada | Long sleepers sleep more and short sleepers sleep less: a comparison of older adults who sleep well | NA | 239 | 55–87  [67.0] | 66.5 |  |  |  |  | Sleep duration (long and short sleepers | Satisfaction with Life Scale |
| *Gellert, 2019  Germany | Profiles of physical activity biographies in relation to life and aging satisfaction in older adults: longitudinal findings | NA | 419 | 60–95  [66.5] | 47 |  |  | PA biographies: How frequently were you consistently physically active in each phase (childhood/adolescence, 30s, 50s, 60s)?” rated on a 6-point Likert-type response format (never, seldom, sometimes, quite often, mostly, and always). Latent class analysis revealed four categories: increasingly active, consistently active, consistently inactive, and decreasingly active. Current PA: “In a normal week, how often do you perform physical exercise?” and “In the last four weeks, how often have you performed physical exercise?” |  |  | Subscale of the Philadelphia Geriatric Center Morale Scale (PGCMS) |
| *Gureje, 2014  Nigeria | Profile and determinants of successful ageing in the Ibadan Study of aging | ISA | 930 | ≥65  [79.0] | 38.9 | Ever smoked? Yes or no | Ever used alcohol? Yes or no | International Physical Activity Questionnaire (IPAQ) grouped into low, moderate and vigorous PA levels. |  |  | Satisfaction With Life Scale (SWLS) |
| Johannesson, 2021  Sweden | Exploring meal frequency and vegetable intake among immigrants 70 years or older in Sweden | PAMC | 131 | 70–84  [74.0] | 49.6 |  |  |  | How often do you have vegetables in your diet? Every meal, At least once per day, Almost every day, About once a week, Almost never, and Never |  | LiSat-11 scale dichotomised into satisfied and dissatisfied for inferential analysis |
| Jung, 2010  South Korea | Factors related to perceived life satisfaction among the elderly in South Korea | NSSLDWE | 3278 | ≥65  [74.3] | 61.7 | Do you smoke? Never, Quitted, or Currently smoking | Do you drink alcohol? Never Quitted, or Currently drinking | Do you exercise? Never, Occasionally, or Regularly | Degree of nutritional diet. |  | Single-item question measured on a 5-point Likert scale |
| *Kang, 2025  China | Impact of physical activity on life satisfaction among middle-aged and older adults in China: A longitudinal national study | CHARLS | 6484 | 45–93  [60.0] | 45.7 |  |  | International Physical Activity Questionnaire (IPAQ) short form categorised into low-, moderate-, and high-intensity PA |  |  | Single-item question “Please think about your life as a whole. How satisfied are you with it?” with options: completely satisfied, very satisfied, somewhat satisfied, not very satisfied, or not at all satisfied. |
| Kolosnitsyna, 2017  Russia | Determinants of life satisfaction in older Russians | RLMS-HSE | 16,369 | ≥55  [68.0] | 74 | Smoker? Yes or No |  |  |  |  | Single-item question "To what extent are you satisfied with your life in general at the present time?" and the answers were: 1) fully satisfied; 2) rather satisfied; 3) both yes and no; 4) less than satisfied; 5) not at all satisfied. |
| †Ku, 2016  Taiwan | Leisure-time physical activity, sedentary behaviors and subjective well-being in older adults: an eight-year longitudinal research | SHLSE | 1268 | ≥70  [75.4] | 49.4 |  | Drinking? Yes or no | Frequency of leisure activity was measured on a scale of five levels (i.e., never, monthly, two or three times per week, one or two times per week, and daily) for four types of activity: walking, group exercise, solitary exercise, and yard/gardening. |  |  | Life Satisfaction Index A (LSIA), 10-item version |
| *Lappan, 2020  United States of America | Longitudinal and reciprocal relationships between psychological well-being and smoking | HRS | 4230 | ≥50  [64.0] | 55 | Do you smoke cigarettes now? Yes or No |  |  |  |  | Satisfaction with Life Scale (SWLS) |
| Lengyel, 2009  Canada | The relationships between food group consumption, self-rated health, and life satisfaction of community-dwelling Canadian older men: The Manitoba Follow-Up Study | MFUS | 1,345 | 70.5–94  [80.2] | 0 |  |  |  | How often do you NOW eat/drink the four food groups: fruit & vegetable, grain product, milk & alternatives, and meat & alternatives? every day, most days, and rarely. |  | Single-item question: “How would you describe your satisfaction with life in general at present?” Excellent, good, fair, poor, or bad. |
| †Liu, 2023  China | Sleep duration and life satisfaction among older people in China: a longitudinal investigation | CHARLS | 4861 | 60–101  [67.1] | 51.45 |  |  |  |  | Self-estimation of average daily sleep hours in the last month. | Single-item question on a Likert scale, 1=completely satisfied to 5=not at all satisfied |
| Maher, 2017  United States of America of America | Daily life satisfaction in older adults as a function of (in) activity | NA | 100 | 60–89  [74.2] | 67 |  |  | Objectively measured using ActivPAL3 activity monitor |  |  | Single-item question “I was satisfied with my life today” 0 (strongly disagree) to 100 (strongly agree). |
| McAuley, 2006  United States of America | Physical activity and quality of life in older adults: influence of health status and self-efficacy | NA | 249 | 59–84  [68.1] | 100 |  |  | Physical Activity Scale for the Elderly (PASE) and Community Healthy Activities Model Program for Seniors physical activity questionnaire (CHAMPS) |  |  | Satisfaction with Life Scale (SWLS) |
| *Morgan, 1998  United Kingdom | Customary physical activity and psychological wellbeing: a longitudinal study | NLSAA | 496; 293 | ≥65  [75.6] |  |  |  | Purposeful walking outside the house or garden at T1 (min per typical day) and Total indoor activities at T2 (min per week) |  |  | Life Satisfaction Index (LSI) at T2 and T3 |
| Ní Mhaoláin, 2012  Ireland | Subjective well-being amongst community-dwelling elders: what determines satisfaction with life? Findings from the Dublin Healthy Aging Study | NA | 466 | ≥65  [75.5] | 55.4 |  |  | “Have you done any exercise in the past 2 weeks?” Yes or No |  | Frequency of disturbed sleep in the past week | Life Satisfaction Index– version A (LSI-A) |
| Papi, 2021  Iran | Relationship between life satisfaction and sleep quality and its dimensions among older adults in the city of Qom, Iran | NA | 679 | 60–92  [70.4] | 68.9 |  |  |  |  | Pittsburgh Sleep Quality Index (PSQI) | Life Satisfaction Index-Z (LSI-Z) |
| Park, 2013  South Korea | Prevalence and predictors of poor sleep quality in Korean older adults | NA | 157 | ≥65  [74.0] | 73.9 |  |  |  |  | Pittsburgh Sleep Quality Index (PSQI). A higher score indicates lower sleep quality | Life Satisfaction Inventory |
| Park, 2014  South Korea | Factors influencing physical activity in older adults | NA | 187 | 57–96  [71.6] | 70.1 |  |  | Physical Activity Survey for the Elderly (PASE) |  |  | Satisfaction with Life Scale (SWLS) |
| Parker, 2008  United States of America | Physical activity measurement in older adults: relationships with mental health | NA | 84 | 55–87  [71.3] | 66.7 |  |  | Physical Activity Scale for the Elderly (PASE); Pedometer (steps/day); Accelerometer (moderate and vigorous physical activity in min/day); and Accelerometer (counts/day) |  |  | Satisfaction With Life Scale (SWLS) |
| Parra-Rizo, 2020  Spain | Satisfaction with life, subjective well-being and functional skills in active older adults based on their level of physical activity practice | NA | 397 | 61–93  [69.7] | 64.7 |  |  | International Physical Activity Questionnaire (IPAQ) |  |  | Life Satisfaction Index-A (LSIA) |
| *Peltzer, 2022  South Africa | Impact of somatic conditions and lifestyle behaviours on depressive symptoms and low life satisfaction among middle-aged and older adult men in South Africa | HAALSI | 2346 | ≥40  [63.1] | 0 | Current tobacco smoking (Yes or No) | Alcohol dependence was measured using the CAGE questionnaire and categorised into Yes or No | General Physical Activity Questionnaire (GPAQ) and classified into low, moderate, and high | Fruit & vegetable intake classified into (0 – 2; 3 – 4; ≥5) |  | Single-item question: “All things considered, how satisfied are you with your life as a whole these days? Use a 0 to 10 scale, where 0 is dissatisfied and 10 is satisfied” |
| †Peng, 2024  China | Lifestyle factors, physical health, and life satisfaction under different changes in depressive symptoms among Chinese community-dwelling older adults: a longitudinal analysis | CHARLS | 1,068 | 60–88  [65.5] | 48.2 | Respondents were classified into non-smokers, former smokers, and current smokers. | Respondents were categorised into non-drinkers, former drinkers, and current drinkers | International Physical Activity Questionnaire (IPAQ). Physical activities were categorised into no physical activity, light-intensity physical activities, moderate physical activities, and vigorous physical activities. |  | Sleep duration was measured by asking the respondents about the average hours of actual sleep they had received for one night during the past month. | Single-item question “Please think about your life as a whole. How satisfied are you with it?” rated on a five-point Likert scale, ranging from 1 = not at all satisfied to 5 = completely satisfied. |
| †Phillips, 2013  United States of America | Physical activity and quality of life in older adults: an 18 month panel analysis | NA | 321; 227 | 50–90,  [63.8; 64.01] | 80.1; 81.5 |  |  | Physical Activity Scale for the Elderly (PASE) |  |  | Satisfaction with Life Scale (SWLS) |
| Phulkerd, 2021  Thailand | Influence of healthy lifestyle behaviors on life satisfaction in the aging population of Thailand: a national population-based survey | NA | 1460 | 60–93  [69.0] | 55.1 | Never smoker, Ex-smoker, and Current smoker | Never drinker, Ex-drinker, and Current drinker | Daily physical activity at a moderate or vigorous level, such as brisk walking, running, aerobics, and competitive games or sports for at least 30 minutes per day, was recorded as ‘yes’ and ‘no.’ | The fruit/vegetable consumption is categorised as “less than 400 grams per day” and “400 grams or above per day.” |  | Scale with Life Satisfaction (SWLS). Categorised as less satisfaction and more satisfaction |
| Rodrigues, 2023  Canada | Sleep problems and psychological well-being: baseline findings from the Canadian Longitudinal Study on Aging | CLSA | 30097 | 45–85  [60.0] | 50 |  |  |  |  | Sleep duration: During the past month, on average, how many hours of actual sleep did you get at night? Answers were categorised as short (< 6 hours), normal (6–8 hours), and long sleep duration (> 8 hours). | Satisfaction with Life Scale (SWLS). The total was categorised into satisfied and dissatisfied. |
| Rodrigues, 2023  Portugal | Motivational correlates, satisfaction with life, and physical activity in older adults: a structural equation analysis | NA | 268 | 65–90  [68.1] |  |  |  | Exercise frequency: “How many days per week do you think you have exercised over the last week?” |  |  | Satisfaction with Life Scale Portuguese version |
| †Shojima, 2024  Japan | Factors contributing to subjective well-being and supporting successful aging among rural Japanese community-dwelling older adults: a cross-sectional and longitudinal study | FESTA | 541 | ≥65  [73.1] | 65.1 | Smoking (Never, Former, Current) | Alcohol drinking (Non-drinker, Drinker) | Steps count (≤6000 steps/day, >6000 steps/day) |  |  | Single-item question: “How satisfied are you with yourself?” Rated on a 5-point response scale ranging from 1 (“not at all”) to 5 (“extremely”). 4 and 5 were classified as satisfied. |
| Siegmund, 2025  United States of America | Physical, social, psychological, and environmental predictors of life satisfaction among older adults | NA | 515 | 65–94  [73.1] | 50 |  |  |  |  | Sleep disturbance was assessed using the Patient-Reported Outcomes Measurement Information System Sleep Disturbance | Satisfaction With Life Scale (SWLS). |
| Skałacka, 2023  Poland | Physical leisure activities and life satisfaction in older adults | NA | 120 | ≥60  [69.0] | 67.5 |  |  | Community Healthy Activities Model Program for Seniors (CHAMPS). The frequency and the intensity (measured as MET) were used for analysis. |  |  | Polish version of WHOQOL-BREF. The overall satisfaction was extracted. |
| Syue, 2022  Taiwan | The associations between physical activity, functional fitness, and life satisfaction among community-dwelling older adults | NA | 623 | ≥65  [73.7] | 60.2 |  |  | International Physical Activity Questionnaire (IPAQ) short form and categorised into low-active, moderate-active, and high-active. |  |  | Single-item question, on an 11-point scale from 0 to 10, was used to evaluate life satisfaction: “In general, how satisfied are you with your life?” |
| Teixeira Vaz, 2019  Brazil | A multilevel model of life satisfaction among old people: individual characteristics and neighborhood physical disorder | BH-SSO | 832 | ≥60  [69.3] | 56.4 | Smoking habits (non-smoker, former smoker, and current smoker), constructed from the following questions: “In your life, have you ever smoked cigarettes?” and “Do you currently smoke cigarettes?” | Alcohol consumption (yes or no), assessed by the question: “Do you drink alcoholic beverages?” | Physical activity (yes or no), assessed by the question: “Do you practice (or have you practised) any physical activity in the past 3 months?” |  |  | Self-Anchoring Ladder Scale (SALS), developed by Cantril. Participants answered: “In relation to satisfaction with your current life, in which rung are you TODAY?” Responses were categorised as satisfied (rungs 6–10) and dissatisfied (rungs 0–5). |
| *Wang, 2024  Taiwan | The combination of physical activity with fruit and vegetable intake associated with life satisfaction among middle-aged and older adults: a 16-year population-based cohort study | TLSA | 3857 | ≥53  [70.3] | 46.6 | Participants were classified as nonsmokers or current/past smokers | Participants responded yes/ no about drinking alcohol: never drink once or twice a week. | Three items assessed daily physical activity's frequency, duration, and intensity. Scores were categorised into low, moderate, and high. | Single-item question assessed the frequency of F&V intake. Response was categorised into low, moderate, and high. |  | Life Satisfaction Index (LSI) |
| White, 2009  United States of America | Physical activity and quality of life in community-dwelling older adults | NA | 321 | ≥50  [63.8] | 80.1 |  |  | Physical Activity Scale for the Elderly (PASE) and Godin Leisure Time Exercise Questionnaire (GLTEQ) |  |  | Satisfaction with Life Scale (SWLS) |
| Wickrama, 2013  United States of America | Linking life dissatisfaction to health behaviors of older african americans through psychological competency and vulnerability | HHUP | 207 | ≥43  [71.1] | 74 |  |  | Yale Physical Activity Survey, with higher scores indicating the performance of more physical activities | Fruit and Vegetable Consumption: A single item assessed the number of fruits and vegetables respondents typically eat daily, with seven response categories ranging from “zero” to “six or more.” Fat Diet: Using a single item, respondents indicated whether their diet over the past 12 months was low (1), medium (2), or high (3) in fat. | Respondents reported the number of minutes it takes for them to fall asleep on an average night | Satisfaction With Life Scale. Sum scores were recoded so that higher scores indicated life dissatisfaction. |
| Wickrama, 2012  United States of America | Life dissatisfaction and eating behaviors among older african americans: the protective role of social support | HHUP | 178 | ≥43  [60.7] | 69 |  |  |  | Fruit and Vegetable Consumption: A single item assessed the number of fruits and vegetables respondents typically eat daily, with seven response categories ranging from “zero” to “six or more.” Fat Diet: Five items rated yes or no (1 = yes, 2 = no), with a high score indicating a high-fat diet. |  | Satisfaction With Life Scale. Sum scores were recoded so that higher scores indicated life dissatisfaction. |
| Withall, 2014  United Kingdom | Objective Indicators of Physical Activity and Sedentary Time and Associations with Subjective Well-Being in Adults Aged 70 and Over | NA | 228 | 70–96  [78.2] | 48.7 |  |  | Physical activity volume (min/day) at moderate-to-vigorous intensity (MVPA) measured using an Actigraph accelerometer |  |  | Satisfaction with Life Scale (SWLS) |
| *Yen, 2024  Taiwan | Leisure participation for mental health promotion in later life: a six-year longitudinal study | PSFD | 1101 | ≥60  [69.2] | 57.1 |  |  | Participants rated the frequency of participation in physical activity on a 5-point scale (never, less than once a month, 1–3 times a month, 1–3 times a week, and >4 times a week |  |  | Single-item question "How satisfied are you with your life?" rated on a 7-point scale |
| You, 2023  South Korea | Religious engagement and successful aging among Korean older adults | KLSA | 4013 | ≥65  [77.1] | 66.8 |  |  | Regular exercise operationalisation was not described |  |  | Satisfaction with Life Scale (SWLS) |
| *Yuan, 2024  China | Bidirectional association between attitudes toward own aging and quality of life in Chinese older adults: A prospective cohort study | CLHLS | 2129 | ≥60,  [76.6] | 49.3 | Current smoking? Yes or no | Excessive drinking? Yes or no | Regular exercise? Yes or no |  | Single-item sleep quality question rated from 1-very bad to 5-very good | Single-item question “How do you feel about your life now?” rated on a 5-point Likert scale from 1 (very good) to 5 (very bad). |
| Zapata-Lamana, 2022  Spain | Health, functional ability, and environmental quality as predictors of life satisfaction in physically active older adults | NA | 397 | 61–93  [69.7] | 64.7 |  |  | International Physical Activity Questionnaire (IPAQ) |  |  | Life Satisfaction Index-A (LSI-A) |
| Zaragoza-Marti, 2018  Spain | Relationship between adherence to the Mediterranean diet and health-related quality of life and life satisfaction among older adults | NA | 351 | ≥60  [71.1] | 57.2 |  |  |  | Mediterranean diet (MD) score is based on the intake of vegetables, legumes, fruits and nuts, cereals, fish, meat and meat products, dairy products, olive oil and red wine. The score ranged from 0 (minimal adherence to the MD) to 9 (maximal adherence to the MD) |  | Satisfaction with Life Scale (SWLS), Spanish version, with each question rated on a 5-point Likert scale; 1 “strongly disagree” to 5 “strongly agree.” |
| Zhi, 2016  China | Associations of sleep duration and sleep quality with life satisfaction in elderly Chinese: The mediating role of depression | RuLAS | 1756 | 70–84  [75.3] | 53.3 |  |  |  |  | Sleep duration: single-item question “How many hours of actual sleep do you obtain at night?”. And categorised into: 6 h per night, 7–8 h per night, and 9 h per night.  Sleep quality: single-item question “In general, would you say your sleep quality is...,” with 4 response options: “well”, “rather well”, “rather poor”, or “poor.” Categorised into sleeping well and sleeping poorly. | Single-item question “How satisfied are you with your current life?” with 5 options: very satisfied, satisfied, fair, unsatisfied, very unsatisfied. Categorised as satisfied and unsatisfied. |
| *Zhu, 2023  China | Reciprocal effects between sleep quality and life satisfaction in older adults: the mediating role of health status | CLHLS | 1856 | [77.22; 80.11] | 48; 48 |  |  |  |  | Single item question “how about the quality of your sleep?” with a score 1 indicating very poor and 5 indicating very good. | Single-item question “how do you rate your life at present?” with a score of 1 representing very bad and 5 representing very good. |

**Dataset:** ACS: Aging in the Community Study. BH-SSO: Belo Horizonte household survey and Systematic Social Observation. CHARLS: China Health and Retirement Longitudinal Study. CLHLS: Chinese Longitudinal Healthy Longevity Survey. CLSA: Canadian Longitudinal Study on Aging. ELSA: English Longitudinal Study of Ageing. FESTA: Frail Elderly in the Sasayama-Tamba Area study. FIBRA: Frailty in Brazilian Older Adults. HAALSI: Health and Ageing in Africa: A Longitudinal Study of an INDEPTH (International Network for the Demographic Evaluation of Populations and their Health) Community in South Africa. HHUP: Health for Hearts United Project. HRS: Health and Retirement Study. HUNT: **H**else**u**ndersøkelsen i **N**ord-**T**røndelag. ISA: Ibadan Study of Aging. KLSA: Korean Longitudinal Study of Aging. MFUS: The Manitoba Follow-up Study. MIDUS: Midlife in the United States. NLSAA: Nottingham Longitudinal Study of Activity and Ageing. NSSLDWE: National Survey on the State of Life and the Desire for Welfare of the Elderly. PAMC: Promoting Aging Migrants’ Capabilities. PROOF: Prognostic Indicator of Cardiovascular and Cerebrovascular Events. PSFD: Panel Study of Family Dynamics. RHS: Rural Health Study. RLMS-HSE: Russia Longitudinal Monitoring Survey of Higher School of Economics. RuLAS: Rugao Longevity and Aging Study. SHLSE: Survey of Health and Living Status of the Elderly. TLSA: Taiwan Longitudinal Survey on Aging.

**Study design:** (no symbol) Cross-sectional analysis. * Longitudinal analysis. † Both Cross-sectional and Longitudinal analyses. **NA:** Not Applicable

**Supplementary File 4:** Summary of results from the included studies (n = 56)

| **First author last name, Year, &**  **Country** | **Title** | **Study design** | **How many years' gap for longitudinal analysis?** | **Analysis type and result** |
| --- | --- | --- | --- | --- |
| Achour, 2011  France | Level of physical activity at the age of 65 predicts successful aging seven years later: the PROOF study | Longitudinal | Seven | BA: Higher physical activity level was significantly associated with higher life satisfaction (r=0.1, p=0.0088).  MA: Higher physical activity level was significantly associated with higher life satisfaction (p=0.0117) when controlled for Sex, family status, peak oxygen uptake, and daily energy expenditure higher than 5 METS. |
| An, 2020  Taiwan | The relationships between physical activity and life satisfaction and happiness among young, middle-aged, and older adults | Cross-sectional |  | MA: Higher physical activity level was significantly associated with higher life satisfaction (β=0.18, p<0.01) when controlled for age, age squared, gender, educational level, marital status, living alone, monthly income, duration of vigorous activity, moderate activity, and walking. |
| Andre, 2017  Norway | Is there an association between food patterns and life satisfaction among Norway's inhabitants ages 65 years and older? | Cross-sectional |  | BA: Individuals in the healthy food cluster had significantly higher life satisfaction than those in the unhealthy food group (χ^2^=62.55, P<0.001). |
| Bae, 2017  United States of America | Physical activity levels and well-being in older adults | Cross-sectional |  | BA: Higher moderate (r=0.07, p<0.05) and light (r=0.1, p<0.01) physical activities were significantly associated with higher life satisfaction, while vigorous physical activity was not (r=0.02, p>0.05).  MA: Only higher light physical activity (β=0.97, p<0.01) predicted higher life satisfaction when adjusted for age, gender, vigorous and moderate Physical activities. |
| Banjare, 2015  India | Factors associated with the life satisfaction amongst the rural elderly in Odisha, India | Cross-sectional |  | MA (Men): Smoking (β=0.102, p>0.05) and alcohol consumption (β=0.357, p>0.05) had no significant influence on life satisfaction when controlled for age, marital status, education, wealth quintile, castle, state of economic dependence, living arrangement, tobacco consumption, activities of daily living, social support, disability, cognitive health, and morbidity status. |
|  |  |  |  | MA (Women): Smoking (β=0.098, p>0.05) and alcohol consumption (β=0.342, p>0.05) had no significant influence on life satisfaction when controlled for age, marital status, education, wealth quintile, castle, state of economic dependence, living arrangement, tobacco consumption, activities of daily living, social support, disability, cognitive health, and morbidity status. |
| Bertelli-Costa, 2021  Brazil | Life satisfaction and participation  among community-dwelling older  adults: Data from the FIBRA study | Cross-sectional |  | BA: Individuals who are active in global (p<0.001) and leisure-time (p<0.001) physical activities had significantly higher life satisfaction than insufficiently active individuals.  MA: Individuals who are active in leisure-time (OR= 1.70, p<0.001) physical activities had significantly higher life satisfaction than insufficiently active individuals when controlled for gender, age, social activity, family income, and occupational, domestic, transportation, and global physical activities. |
| Bourque, 2005  Canada | Contextual effects on life satisfaction of older men and women | Cross-sectional |  | BA: Higher physical activity level was significantly associated with higher life satisfaction in men (r=0.19, p<0.001) and women (r=0.24, p<0.001). |
| Bourque, 2007  Canada | Self-reported sensory impairment and life satisfaction in older French-speaking adults | Cross-sectional |  | BA: Lower physical activity level was significantly associated with higher life satisfaction (r=-0.2, p<0.001).  MA: Physical activity was not a significant predictor of life satisfaction (β=-0.05, p>0.05) when adjusted for age, sex, education, income, number of chronic illnesses, functional limitations, story recall, social support, social activity, perceived control, vision, hearing, and vision plus hearing |
| Caligiuri, 2012  Canada | Changes in food group consumption and associations with self-rated diet, health, life satisfaction, and mental and physical functioning over 5 years in very old Canadian men: The Manitoba Follow-Up Study | Longitudinal | Five | BA: Individuals who improved their diet in all food groups (P>0.5) did not have a significant increase in life satisfaction. |
| Cho, 2023  United States of America | Older adults’ advance aging and life satisfaction levels: effects of lifestyles and health capabilities | Cross-sectional |  | BA: Individuals who engaged in regular physical activity had significantly higher life satisfaction than those who did not (F=10.69, P=0.001). There was no significant influence of smoking (F=0.68, p=0.410), drinking alcohol (F=0.74, p=0.390), and diet (F=0.81, P=0.370). |
| Colsher, 1990  United States of America | Elderly men with histories of heavy drinking: correlates and consequence | Cross-sectional |  | BA: Heavy drinkers had significantly lower life satisfaction than non-heavy drinkers (p<0.01), both at baseline and follow-up. |
| Crawford-Achour, 2014  France | Can subjective sleep quality, evaluated at the age of 73, have an influence on successful aging? The PROOF study | Cross-sectional |  | BA: Quality sleep was associated with higher life satisfaction (r=-0.19, p<0.001).  MA: Quality sleep was associated with higher life satisfaction (B=-0.109, p=0.001) when adjusted for gender, educational level, and apnea-hypopnea index. |
| Di Gessa, 2023  United Kingdom | Health behaviors and mental health during the COVID-19 pandemic: evidence from the English Longitudinal Study of Aging | Cross-sectional |  | MA: Drinking more alcohol (B=-0.39, P<0.001), less physical activity (B=-0.44, p<0.001), and less sleep duration (B=-0.80, p<0.001) significantly decreased life satisfaction when controlled for age, sex, ethnicity, education, pre-pandemic wealth, living arrangements, employment status, have enough food, frequency of contact with family and friends, COVID-19 negative experiences and vulnerability, disability, pre-pandemic mental health. |
|  |  | Longitudinal | Six months | MA: Increased alcohol drinking (B=-0.23, P<0.005), decreased physical activity (B=-0.24, p<0.01), and reduced sleep duration (B=-0.47, p<0.001) significantly decreased life satisfaction when controlled for age, sex, ethnicity, education, pre-pandemic wealth, living arrangements, employment status, have enough food, frequency of contact with family and friends, COVID-19 negative experiences and vulnerability, disability, pre-pandemic mental health. |
| Fichten, 2004  Canada | Long sleepers sleep more and short sleepers sleep less: a comparison of older adults who sleep well | Cross-sectional |  | BA: Long sleepers did not have a significantly higher life satisfaction than short sleepers (t=0.12, p>0.05). |
| Gellert, 2019  Germany | Profiles of physical activity biographies in relation to life and aging satisfaction in older adults: longitudinal findings | Longitudinal | One | MA: Consistently active individuals had a significantly higher life satisfaction (β=0.17, p=0.028) when adjusted for current physical activity. |
| Gureje, 2014  Nigeria | Profile and determinants of successful ageing in the Ibadan Study of aging | Longitudinal | Five | MA: There was no significant influence of smoking (OR=1.2, p=0.129), alcohol drinking (OR=1.1, p=0.411), and physical activity (OR=0.8, p=0.337) when controlled for sex, age, education, residence, economic status, contact with family, contact with friends, participation in household activity, participation in community activity, and self-rated health. |
| Johannesson, 2021  Sweden | Exploring meal frequency and vegetable intake among immigrants 70 years or older in Sweden | Cross-sectional |  | BA: Fruit and vegetable diet was not significantly associated with life satisfaction (OR: 1.35, p=0.09).  MA: Fruit and vegetable diet was not significantly associated with life satisfaction (OR: 1.35, p=0.09) when adjusted for gender, age, education, living alone, level of physical activity, smoking, and alcohol. |
| Jung, 2010  South Korea | Factors related to perceived life satisfaction among the elderly in South Korea | Cross-sectional |  | MA (Men): Higher alcohol drinking (B=0.073, p<0.01) and higher degree of nutritional diet (B=0.107, p<0.001) were significantly associated with higher life satisfaction, while there were no significant effects of smoking (B=-0.007, p>0.05) and physical activity (B=-0.030, p>0.05) when controlled for age, marital status, educational level, employment status, economic situation, type of household, existence or not of persons requiring care, reception or not of health checkup, health condition compared with the same age group, number of chronic diseases, capability of activities of daily living, cognitive function, perception on the age of the elderly, frequency of contact with children, and performance or not of social activities. |
|  |  |  |  | MA (Women): Higher degree of nutritional diet (B=0.145, p<0.001) was significantly associated with higher life satisfaction, while there were no significant effects of smoking (B=0.015, p>0.05), alcohol drinking (B=-0.004, p>0.05) and physical activity (B=0.004, p>0.05) when controlled for age, marital status, educational level, employment status, economic situation, type of household, existence or not of persons requiring care, reception or not of health checkup, health condition compared with the same age group, number of chronic diseases, capability of activities of daily living, cognitive function, perception on the age of the elderly, frequency of contact with children, and performance or not of social activities. |
| Kang, 2025  China | Impact of physical activity on life satisfaction among middle-aged and older adults in China: A longitudinal national study | Longitudinal | Three | BA: Low-intensity physical activity group experienced significantly lower levels of life satisfaction (OR=0.872, p=0.037).  MA: Low-intensity physical activity group experienced significantly lower levels of life satisfaction (OR=0.823, p=0.004) when adjusted for age, sex, education, marital status, residence, alcohol consumption, sleep duration, BMI, number of chronic diseases, depressive symptoms, and self-rated health. |
| Kolosnitsyna, 2017  Russia | Determinants of life satisfaction in older Russians | Cross-sectional |  | MA: Smoking did not significantly influence life satisfaction (p>0.05) when adjusted for age, age squared, type of settlement, health status, smoking, education, income, job, social status, marital status, and children. |
| Ku, 2016  Taiwan | Leisure-time physical activity, sedentary behaviors and subjective well-being in older adults: an eight-year longitudinal research | Cross-sectional |  | BA: Higher physical activity was significantly associated with higher life satisfaction (rho=0.29, p<0.001). |
|  |  | Longitudinal | Eight | BA: Higher physical activity was significantly associated with higher life satisfaction (rho=0.21, p<0.001).  MA: Higher physical activity was significantly associated with higher life satisfaction (β=0.12, p<0.001), but no significant influence of alcohol drinking (β=0.002, p=0.89) when adjusted for leisure time sedentary behaviour, sex, age, education, number of chronic diseases, difficulty in ADL, depressive symptoms, and cognitive decline. |
| Lappan, 2020  United States of America | Longitudinal and reciprocal relationships between psychological well-being and smoking | Longitudinal | Four | MA: Being a smoker was significantly associated with higher life satisfaction (β-0.25, p<0.001) when controlled for gender, race, age, and years of education. |
| Lengyel, 2009  Canada | The relationships between food group consumption, self-rated health, and life satisfaction of community-dwelling Canadian older men: The Manitoba Follow-Up Study | Cross-sectional |  | BA: Eating fruit & vegetables daily (OR=3.71, p=0.016) was significantly associated with higher life satisfaction, but no association for other food groups (p>0.005).  MA: No food groups were associated with life satisfaction (p>0.05) when controlled for age, ever smoked, prior ischemic heart disease, marital status, living arrangements, mental, and physical function. |
| Liu, 2023  China | Sleep duration and life satisfaction among older people in China: a longitudinal investigation | Cross-sectional |  | BA: Long sleep duration was significantly associated with higher life satisfaction (r=0.09, p<0.001). |
|  |  | Longitudinal | Seven | MA: Long sleep duration was significantly associated with higher life satisfaction (r=0.1, p<0.001). |
| Maher, 2017  United States of America of America | Daily life satisfaction in older adults as a function of (in) activity | Cross-sectional |  | MA: No significant influence of physical activity on life satisfaction (B=0.01,p>0.05) when adjusted for between-person factors: usual sedentary behavior, usual physical activity, usual musculoskeletal symptoms, usual gastrointestinal symptoms, usual cold/flu symptoms, usual cardiorespiratory symptoms, sex, age, and BMI. |
| McAuley, 2006  United States of America | Physical activity and quality of life in older adults: influence of health status and self-efficacy | Cross-sectional |  | BA: Significant association between higher physical activity and higher life satisfaction (r=0.21, p<0.05). |
| Morgan, 1998  United Kingdom | Customary physical activity and psychological wellbeing: a longitudinal study | Longitudinal | Four | MA: Higher time spent walking at baseline (T1) was significantly associated with higher life satisfaction at the first follow-up (T2) (β=0.125, p<0.001) when controlled for life satisfaction (T1), social engagement (T1 & T2), health index (T2), age, marital status (T1 & T2), and total indoor activities (T1 & T2). |
|  |  |  | Eight | MA: Higher total indoor activity (T2) was significantly associated with higher life satisfaction at the second follow-up (T3) (β=-0.102, p=0.041) when adjusted for life satisfaction score (T1 & T2), social engagement (T2 & T3), health index (T3), and sex. |
| Ní Mhaoláin, 2012  Ireland | Subjective well-being amongst community-dwelling elders: what determines satisfaction with life? Findings from the Dublin Healthy Aging Study | Cross-sectional |  | BA: Participating in physical activity (rho=0.208, p<0.001) and lower frequency of sleep disturbance (rho=-0.269, p<0.001) were significantly associated with higher life satisfaction.  MA: Participating in physical activity (B=1.13, p=0.005) was significantly associated with higher life satisfaction, but no influence of disturbed sleep frequency (B=-0.155, p=0.317) when controlled for instrumental activities of daily living, physical activity, driving, depression, loneliness, exhaustion, neuroticism, extroversion, age of leaving school, mental state, marital status, and age. |
| Papi, 2021  Iran | Relationship between life satisfaction and sleep quality and its dimensions among older adults in the city of Qom, Iran | Cross-sectional |  | BA: Quality sleep was associated with higher life satisfaction (p<0.001). |
| Park, 2013  South Korea | Prevalence and predictors of poor sleep quality in Korean older adults | Cross-sectional |  | BA: Quality sleep was significantly associated with higher life satisfaction (r=-0.52, p<0.01). |
| Park, 2014  South Korea | Factors influencing physical activity in older adults | Cross-sectional |  | BA: Higher physical activity was significantly associated with higher life satisfaction (rho=0.163, p<0.05). |
| Parker, 2008  United States of America | Physical activity measurement in older adults: relationships with mental health | Cross-sectional |  | BA: No significant association between life satisfaction and physical activity measured using the Physical Activity Scale for the Elderly (r=0.055, p=0.625) and accelerometer for moderate and vigorous physical activity in min/day (r=0.22,p=0.051). There was a significant association using pedometer (steps/day) (r=0.426, p<0.001) and Accelerometer (counts/day) (r=0.299, p=0.007). |
| Parra-Rizo, 2020  Spain | Satisfaction with life, subjective well-being and functional skills in active older adults based on their level of physical activity practice | Cross-sectional |  | BA: No significant difference in life satisfaction among individuals who engage in high, moderate, and low physical activities (F=2.977; η2=0.015, p=0.052). |
| Peltzer, 2022  South Africa | Impact of somatic conditions and lifestyle behaviours on depressive symptoms and low life satisfaction among middle-aged and older adult men in South Africa | Longitudinal | Four | BA: Low incident life satisfaction was significantly associated with a high physical activity (OR=0.59, p<0.001), but not with smoking (OR=0.85, p>0.05), alcohol dependence (OR=0.98, p>0.05), fruit & vegetable intake (OR=0.73, p>0.05). Low persistent life satisfaction was significantly associated with being a smoker (OR=1.51, p<0.01) and a high physical activity (OR=0.7, p<0.01), but not with alcohol dependence (OR=1.65, p>0.05), fruit & vegetable intake (OR=0.88, p>0.05).  MA: Low incident life satisfaction was significantly associated with a high physical activity (OR=0.68, p<0.001) when controlled for age, education, cardiovascular disease, anemia, and kidney disease. Low persistent life satisfaction was significantly associated with being a smoker (OR=1.64, p<0.001) and a high physical activity (OR=0.73, p<0.02) when adjusted for age, education, and HIV. |
| Peng, 2024  China | Lifestyle factors, physical health, and life satisfaction under different changes in depressive symptoms among Chinese community-dwelling older adults: a longitudinal analysis | Longitudinal | Seven | MA: No significant influence of smoking (B=0.626, p>0.05), alcohol drinking (B=-0.489, p>0.05), physical activity (B=-0.033, p>0.05), and sleep (B=0.008, p>0.05) on life satisfaction when adjusted for age, sex, marital status, education, self-rated financial status, status of receiving pension, multimorbidity, and BMI. |
|  |  | Cross-sectional |  | MA: Higher life satisfaction was significantly associated with being a current smoker (B=-0.112, p<0.05), but not with alcohol drinking (B=0.080, p>0.05), physical activity (B=-0.030, p>0.05), and sleep duration (B=0.006, p>0.05) when adjusted for age, sex, marital status, education, self-rated financial status, status of receiving pension, multimorbidity, and BMI. |
| Phillips, 2013  United States of America | Physical activity and quality of life in older adults: an 18 month panel analysis | Longitudinal | One and a half | BA: No significant association between physical activity and life satisfaction (r=0.01, p>0.05). |
|  |  | Cross-sectional |  | BA: Higher physical activity was significantly associated with higher life satisfaction (r=0.14, p<0.05). |
| Phulkerd, 2021  Thailand | Influence of healthy lifestyle behaviors on life satisfaction in the aging population of Thailand: a national population-based survey | Cross-sectional |  | BA: Higher life satisfaction was associated with being physically active (OR=1.648, p<0.001) and higher fruit & vegetable consumption (OR=1.358, p=0.023), but not with smoking (OR=0.928, p=0.678) and alcohol drinking (OR=0.954, p=0.772).  MA: Higher life satisfaction was associated with being physically active (OR=1.662, p<0.001) but not with fruit & vegetable consumption (OR=1.309, p=0.056), smoking (OR=0.745, p=0.212) and alcohol drinking (OR=0.949, p=0.786) when controlled for sex, age, marital status, education, residence, denture wearing, and chronic disease |
| Rodrigues, 2023  Canada | Sleep problems and psychological well-being: baseline findings from the Canadian Longitudinal Study on Aging | Cross-sectional |  | BA: Abnormal sleep duration was significantly associated with a higher prevalence of life dissatisfaction (PR=1.46–2.19, p<0.05).  MA: Shorter sleep duration was significantly associated with a higher prevalence of life dissatisfaction (PR=1.38, P<0.05) when controlled for Sex, age, ethnicity, residence, household income, employment, education, marital status, provided caregiving assistance, alcohol consumption, smoking status, physical activity, fruit/vegetable consumption, usually experiencing pain/discomfort, social support availability, and number of chronic diseases. |
| Rodrigues, 2023  Portugal | Motivational correlates, satisfaction with life, and physical activity in older adults: a structural equation analysis | Cross-sectional |  | No significant association between physical activity and life satisfaction (r=0.05, p>0.05). |
| Shojima, 2024  Japan | Factors contributing to subjective well-being and supporting successful aging among rural Japanese community-dwelling older adults: A cross-sectional and longitudinal study | Cross-sectional |  | MA: No significant association between the prevalence of life satisfaction and smoking (PR=0.98, p=0.957), alcohol drinking (PR=1.14, p=0.259), and physical activity (PR=1.09, p=0.454) when controlled for sex, age group, education, economic status, number of comorbidities, frailty, satisfaction with access to health services, and having a higher-level functional capacity. |
|  |  | Longitudinal | Two | MA: No significant association between the prevalence of life satisfaction and smoking (PR=1.30, p=0.732), alcohol drinking (PR=0.80, p=0.498), and physical activity (PR=0.89, p=0.707) when controlled for sex, age group, education, economic status, number of comorbidities, frailty, satisfaction with access to health services, and having a higher-level functional capacity |
| Siegmund, 2025  United States of America | Physical, social, psychological, and environmental predictors of life satisfaction among older adults | Cross-sectional |  | BA: Sleep disturbance was significantly negatively associated with life satisfaction (β=-0.25, p<0.001).  MA: Sleep disturbance was significantly negatively associated with life satisfaction (β=-0.07, p=0.017) when adjusted for Physical ability, depression, social isolation, living alone, and social vulnerability. |
| Skałacka, 2023  Poland | Physical leisure activities and life satisfaction in older adults | Cross-sectional |  | BA: Higher frequency (rho=0.33, p=0.001) and intensity (rho=0.55, p=0.001) of physical activity were associated with a higher life satisfaction. |
| Syue, 2022  Taiwan | The associations between physical activity, functional fitness, and life satisfaction among community-dwelling older adults | Cross-sectional |  | BA: Higher physical activity was associated with a higher life satisfaction (rho=0.14, p<0.001).  MA: Higher physical activity was associated with a higher life satisfaction (B=0.41, p<0.05) when adjusted for age, sex, educational level, marital status, living accommodation status, arm curl test, chair stand test, back scratch test, chair sit and reach test, 2-minute step test, single leg stand test, and seated up-and-go test. |
| Teixeira Vaz, 2019  Brazil | A multilevel model of life satisfaction among old people: individual characteristics and neighborhood physical disorder | Cross-sectional |  | BA: The prevalence of life satisfaction was significantly higher among those who never smoked (PR=1.2, p=0.041) and those who engage in physical activity (PR=1.3, p=0.01), but not associated with drinking (PR=1, p=0.921).  MA: The prevalence of life satisfaction was significantly higher among those who engage in physical activity (PR=1.11, p=0.023) when adjusted for age, sex, family income, religion participation, physical activity, self-rated health, walking environment, safety, garbage collection and school services. |
| Wang, 2024  Taiwan | The combination of physical activity with fruit and vegetable intake associated with life satisfaction among middle-aged and older adults: a 16-year population-based cohort study | Longitudinal | Sixteen | MA: Higher life satisfaction was associated with being a nonsmoker (OR=0.76, p<0.05), having high physical activity (OR=1.74, p<0.05), and having high frequency of fruit & vegetable intake (OR=2.07, p<0.05), but not with drinking (OR=1.15, p>0.05) when adjusted for sex, age, education, marital status, hypertension, heart disease, diabetes, stroke, cancer, and tea consumption. |
| White, 2009  United States of America | Physical activity and quality of life in community-dwelling older adults | Cross-sectional |  | BA: No significant association between life satisfaction and physical activity when measured using the Physical Activity Scale for the Elderly (r=0.05, p>0.05), but a significant association when measured using the Godin Leisure Time Exercise Questionnaire (r=0.14, p<0.01). |
| Wickrama, 2013  United States of America | Linking life dissatisfaction to health behaviors of older African Americans through psychological competency and vulnerability | Cross-sectional |  | BA: Life dissatisfaction was significantly negatively linked to physical activity (r=-0.17, p<0.05), fruit & vegetable consumption (r=-0.16, p<0.05), and positively linked to fat consumption (r=0.17, p<0.05), but not with sleep (r=0.12, p>0.05). |
| Wickrama, 2012  United States of America | Life dissatisfaction and eating behaviors among older African Americans: the protective role of social support | Cross-sectional |  | BA: Life dissatisfaction was significantly negatively linked to fruit & vegetable consumption (r=-0.19, p<0.05), and positively linked to fat consumption (r=0.23, p<0.05). |
| Withall, 2014  United Kingdom | Objective indicators of physical activity and sedentary time and associations with subjective well-being in adults aged 70 and over | Cross-sectional |  | No significant association between physical activity and life satisfaction (r=0.123, p>0.05). |
| Yen, 2024  Taiwan | Leisure participation for mental health promotion in later life: a six-year longitudinal study | Longitudinal | Six | MA: Higher frequency of physical activity was significantly associated with higher life satisfaction (β=0.08, p<0.001) when controlled for age, sex, education level, retirement, marital status, the number of people living together, passive activities, social activities, and time. |
| You, 2023  South Korea | Religious engagement and successful aging among korean older adults | Cross-sectional |  | BA: Regular exercise was significantly associated with higher life satisfaction (r=0.19, p<0.05).  MA: Regular exercise was significantly associated with higher life satisfaction (β=0.11, p<0.05) when adjusted for Age, gender, marital status, education, health conditions, independence, social contacts, social engagement, leisure participation, depression, loneliness, and religious engagement. |
| Yuan, 2024  China | Bidirectional association between attitudes toward own aging and quality of life in Chinese older adults: A prospective cohort study | Longitudinal | Seven | MA: Life satisfaction was significantly negatively associated with non-regular exercise (β=-0.062, p<0.01) and positively associated with sleep quality (β=0.123, p<0.001) but not with smoking (β=0.008, p>0.05) and excessive drinking (β=-0.025, p>0.05) when adjusted for life satisfaction at other time points, attitude towards own aging, age, sex, marital status, residential area, living arrangement, visual impairment, hearing impairment, toothache, jaw joint pain, stand up alone, hunchbacked, feel not-well, social activity, and indoor activity. |
| Zapata-Lamana, 2022  Spain | Health, functional ability, and environmental quality as predictors of life satisfaction in physically active older adults | Cross-sectional |  | BA: Higher life satisfaction was significantly associated with higher life satisfaction (rho=0.121, p<0.05). |
| Zaragoza-Marti, 2018  Spain | Relationship between adherence to the Mediterranean diet and health-related quality of life and life satisfaction among older adults | Cross-sectional |  | MA (Men): No significant association between Mediterranean diet and life satisfaction (β=-0.10, p=0.31) when controlled for age, hours of physical activity, educational level, body mass index, blood cholesterol, blood glucose levels and blood pressure levels. |
|  |  |  |  | MA (Women): Mediterranean diet was significantly associated with high life satisfaction (β=0.22, p=0.005) when controlled for age, hours of physical activity, educational level, body mass index, blood cholesterol, blood glucose levels and blood pressure levels. |
| Zhi, 2016  China | Associations of sleep duration and sleep quality with life satisfaction in elderly Chinese: The mediating role of depression | Cross-sectional |  | BA: Short sleep duration (OR=3.06, p<0.001) and poor sleep quality (OR=3.30, p<0.001) were significantly associated with life dissatisfaction.  MA: Short sleep duration (OR=2.44, p<0.001) and poor sleep quality (OR=2.66, p<0.001) were significantly associated with life dissatisfaction when controlled for age, gender, occupation, marital status, education level, ADL and depression. |
| Zhu, 2023  China | Reciprocal effects between sleep quality and life satisfaction in older adults: the mediating role of health status | Longitudinal | Three | MA: Quality sleep was significantly associated with high life satisfaction (β=0.072, p<0.001) when adjusted for age, gender, marriage, physical exercise, social security, and cognitive ability. |
|  |  |  | Four | MA: Quality sleep was significantly associated with high life satisfaction (β=0.077, p<0.001) when adjusted for age, gender, marriage, physical exercise, social security, and cognitive ability. |

**Analysis type:** BA: Bivariate Analysis. MA: Multivariate Analysis

**Supplementary File 5:** Risk of Bias Assessment for all studies (n = 56)

| **First author last name, Year, & Country** | **Title** | **Joanna Briggs Institute’s appraisal checklist for analytic cross-sectional studies** | | | | | | | | | |
| --- | --- | --- | --- | --- | --- | --- | --- | --- | --- | --- | --- |
|  |  | **Item 1** | **Item 2** | **Item 3** | **Item 4** | **Item 5** | **Item 6** | **Item 7** | **Item 8** | **Score** | **Risk** |
| Achour, 2011  France | Level of physical activity at the age of 65 predicts successful aging seven years later: the PROOF Study | Yes | Yes | Yes | Yes | Yes | Yes | Yes | Yes | 8 | Low |
| An, 2020  Taiwan | The relationships between physical activity and life satisfaction and happiness among young, middle-aged, and older adults | Yes | Yes | Yes | Yes | Yes | Yes | Yes | Yes | 8 | Low |
| Andre, 2017  Norway | Is there an association between food patterns and life satisfaction among Norway's inhabitants ages 65 years and older? | Yes | Yes | Yes | Yes | No | No | Yes | Yes | 6 | Low |
| Bae, 2017  United States of America | Physical activity levels and well-being in older adults | Yes | Yes | Yes | Yes | Yes | Yes | Yes | Yes | 8 | Low |
| Banjare, 2015  India | Factors associated with the life satisfaction amongst the rural elderly in Odisha, India | Yes | Yes | Yes | Yes | Yes | Yes | Yes | Yes | 8 | Low |
| Bertelli-Costa, 2021  Brazil | Life satisfaction and participation  among community-dwelling older  adults: data from the FIBRA study | Yes | Yes | Yes | Yes | Yes | Yes | Yes | Yes | 8 | Low |
| Bourque, 2005  Canada | Contextual effects on life satisfaction of older men and women | Yes | Yes | Unclear | Yes | Yes | Yes | Yes | Unclear | 6 | Low |
| Bourque, 2007  Canada | Self-reported sensory impairment and life satisfaction in older French-speaking adults | Yes | Yes | Unclear | Yes | Yes | Yes | Yes | Yes | 7 | Low |
| Caligiuri, 2012  Canada | Changes in food group consumption and associations with self-rated diet, health, life satisfaction, and mental and physical functioning over 5 years in very old Canadian men: The Manitoba Follow-Up Study | Yes | Yes | Yes | Yes | No | No | Yes | Yes | 6 | Low |
| Cho, 2023  United States of America | Older adults’ advance aging and life satisfaction levels: effects of lifestyles and health capabilities | Unclear | Yes | Yes | Yes | Yes | Yes | Yes | Yes | 7 | Low |
| Colsher, 1990  United States of America | Elderly men with histories of heavy drinking: correlates and consequence | Yes | Yes | Yes | Unclear | Yes | No | Unclear | Unclear | 4 | Moderate |
| Crawford-Achour, 2014  France | Can subjective sleep quality, evaluated at the age of 73, have an influence on successful aging? The PROOF study | No | Yes | Yes | Yes | Yes | Yes | Yes | Yes | 7 | Low |
| Di Gessa, 2023  United Kingdom | Health behaviors and mental health during the covid-19 pandemic: evidence from the English Longitudinal Study of Aging | Yes | Yes | Yes | Yes | Yes | Yes | Yes | Yes | 8 | Low |
| Fichten, 2004  Canada | Long sleepers sleep more and short sleepers sleep less: a comparison of older adults who sleep well | Yes | Yes | Yes | Yes | Yes | Yes | Yes | Yes | 8 | Low |
| Gellert, 2019  Germany | Profiles of physical activity biographies in relation to life and aging satisfaction in older adults: longitudinal findings | Yes | Yes | Unclear | Yes | Yes | Yes | Yes | Yes | 7 | Low |
| Gureje, 2014  Nigeria | Profile and determinants of successful ageing in the Ibadan Study of aging | Yes | Yes | Yes | Yes | Yes | Yes | Yes | Yes | 8 | Low |
| Johannesson, 2021  Sweden | Exploring meal frequency and vegetable intake among immigrants 70 years or older in Sweden | Yes | Yes | Yes | Yes | Yes | Yes | Yes | Yes | 8 | Low |
| Jung, 2010  South Korea | Factors related to perceived life satisfaction among the elderly in South Korea | Yes | Yes | Unclear | Unclear | Yes | Yes | Unclear | Yes | 5 | Moderate |
| Kang, 2025  China | Impact of physical activity on life satisfaction among middle-aged and older adults in China: A longitudinal national study | Yes | Yes | Yes | Yes | Yes | Yes | Yes | Yes | 8 | Low |
| Kolosnitsyna, 2017  Russia | Determinants of life satisfaction in Older Russians | Yes | Yes | Yes | Yes | Yes | Yes | Yes | Unclear | 7 | Low |
| Ku, 2016  Taiwan | Leisure-time physical activity, sedentary behaviors and subjective well-being in older adults: an eight-year longitudinal research | Yes | Yes | Yes | Yes | Yes | Yes | Yes | Yes | 8 | Low |
| Lappan, 2020  United States of America | Longitudinal and reciprocal relationships between psychological well-being and smoking | Unclear | Unclear | Yes | Yes | Yes | Yes | Yes | Yes | 6 | Low |
| Lengyel, 2009  Canada | The relationships between food group consumption, self-rated health, and life satisfaction of community-dwelling Canadian older men: The Manitoba Follow-Up Study | Yes | Yes | Yes | Unclear | Yes | Yes | Unclear | Yes | 6 | Low |
| Liu, 2023  China | Sleep duration and life satisfaction among older people in China: a longitudinal investigation | Yes | Yes | Yes | Unclear | Yes | Yes | Yes | Yes | 7 | Low |
| Maher, 2017  United States of America | Daily life satisfaction in older adults as a function of (in) activity | Yes | Yes | Yes | Yes | Yes | Yes | Yes | Yes | 8 | Low |
| McAuley, 2006  United States of America | Physical activity and quality of life in older adults: influence of health status and self-efficacy | Yes | Unclear | Yes | Yes | Yes | Yes | Yes | Yes | 7 | Low |
| Morgan, 1998  United Kingdom | Customary physical activity and psychological wellbeing: a longitudinal study | Unclear | Yes | Unclear | Yes | Yes | Yes | Yes | Yes | 6 | Low |
| Ni Mhaolain, 2012  Ireland | Subjective well-being amongst community-dwelling elders: what determines satisfaction with life? Findings from the Dublin Healthy Aging Study | Yes | Yes | Unclear | Yes | Yes | Yes | Yes | Yes | 7 | Low |
| Papi, 2021  Iran | Relationship between life satisfaction and sleep quality and its dimensions among older adults in city of Qom, Iran | Yes | Yes | Yes | Yes | Unclear | Unclear | Yes | Unclear | 5 | Moderate |
| Park, 2013  South Korea | Prevalence and predictors of poor sleep quality in Korean older adults | Yes | Yes | Yes | Yes | Yes | Yes | Yes | Yes | 8 | Low |
| Park, 2014  South Korea | Factors influencing physical activity in older adults | Yes | Yes | Yes | Yes | Yes | Yes | Yes | Yes | 8 | Low |
| Parker, 2008  United States of America | Physical activity measurement in older adults: relationships with mental health | Yes | Yes | Yes | Yes | No | No | Yes | Yes | 6 | Low |
| Parra-Rizo, 2020  Spain | Satisfaction with life, subjective well-being and functional skills in active older adults based on their level of physical activity practice | Yes | Yes | Yes | Yes | No | No | Yes | Yes | 6 | Low |
| Peltzer, 2022  South Africa | Impact of somatic conditions and lifestyle behaviours on depressive symptoms and low life satisfaction among middle-aged and older adult men in South Africa | Yes | Yes | Yes | Yes | Yes | Yes | Yes | Yes | 8 | Low |
| Peng, 2024  China | Lifestyle factors, physical health, and life satisfaction under different changes in depressive symptoms among Chinese community-dwelling older adults: A longitudinal analysis | Yes | Yes | Yes | Yes | Yes | Yes | Yes | Yes | 8 | Low |
| Phillips, 2013  United States of America | Physical activity and quality of life in older adults: an 18 month panel analysis | Yes | Yes | Yes | Yes | Yes | Yes | Yes | Yes | 8 | Low |
| Phulkerd, 2021  Thailand | Influence of healthy lifestyle behaviors on life satisfaction in the aging population of Thailand: a national population-based survey | Yes | Yes | Yes | Yes | Yes | Yes | Yes | Yes | 8 | Low |
| Rodrigues, 2023  Canada | Sleep problems and psychological well-being: baseline findings from the Canadian Longitudinal Study on Aging | Yes | Yes | Yes | Yes | Yes | Yes | Yes | Yes | 8 | Low |
| Rodrigues, 2023  Portugal | Motivational correlates, satisfaction with life, and physical activity in older adults: a structural equation analysis | Yes | Unclear | Yes | Yes | Yes | Yes | Yes | Yes | 7 | Low |
| Shojima, 2024  Japan | Factors contributing to subjective well-being and supporting successful aging among rural Japanese community-dwelling older adults: A cross-sectional and longitudinal study | Yes | Yes | Yes | Yes | Yes | Yes | Yes | Yes | 8 | Low |
| Siegmund, 2025  United States of America | Physical, social, psychological, and environmental predictors of life satisfaction among older adults | Yes | Yes | Yes | Yes | Yes | Yes | Yes | Yes | 8 | Low |
| Skałacka, 2023  Poland | Physical leisure activities and life satisfaction in older adults | Yes | Yes | Yes | Yes | No | No | Yes | Yes | 6 | Low |
| Syue, 2022  Taiwan | The associations between physical activity, functional fitness, and life satisfaction among community-dwelling older adults | Yes | Yes | Yes | Yes | Yes | Yes | Yes | Yes | 8 | Low |
| Teixeira Vaz, 2019  Brazil | A multilevel model of life satisfaction among old people: individual characteristics and neighborhood physical disorder | Yes | Yes | Yes | Yes | Yes | Yes | Yes | Yes | 8 | Low |
| Wang, 2024  Taiwan | The combination of physical activity with fruit and vegetable intake associated with life satisfaction among middle-aged and older adults: a 16-year population-based cohort study | Yes | Yes | Yes | Yes | Yes | Yes | Yes | Yes | 8 | Low |
| White, 2009  United States of America | Physical activity and quality of life in community dwelling older adults | Unclear | Yes | Yes | Yes | Yes | Yes | Yes | Yes | 7 | Low |
| Wickrama, 2013  United States of America | Linking life dissatisfaction to health behaviors of older African Americans through psychological competency and vulnerability | Yes | Yes | Yes | Yes | No | No | Yes | Yes | 6 | Low |
| Wickrama, 2012  United States of America | Life dissatisfaction and eating behaviors among older African Americans: the protective role of social support | Yes | Yes | Yes | Yes | No | No | Yes | Yes | 6 | Low |
| Withall, 2014  United Kingdom | Objective indicators of physical activity and sedentary time and associations with subjective well-being in adults aged 70 and over | Yes | Yes | Yes | Yes | Yes | Yes | Yes | Unclear | 7 | Low |
| Yen, 2024  Taiwan | Leisure participation for mental health promotion in later life: a six-year longitudinal study | Yes | Yes | Yes | Yes | Yes | Yes | Yes | Yes | 8 | Low |
| You, 2023  South Korea | Religious engagement and successful aging among Korean older adults | Yes | Yes | Unclear | Yes | Yes | Yes | Yes | Yes | 7 | Low |
| Yuan, 2024  China | Bidirectional association between attitudes toward own aging and quality of life in Chinese older adults: A prospective cohort study | Yes | Yes | Yes | Yes | Yes | Yes | Yes | Yes | 8 | Low |
| Zapata-Lamana, 2022  Spain | Health, functional ability, and environmental quality as predictors of life satisfaction in physically active older adults | Yes | Yes | Yes | Yes | Yes | Yes | Yes | Yes | 8 | Low |
| Zaragoza-Marti, 2018  Spain | Relationship between adherence to the mediterranean diet and health-related quality of life and life satisfaction among older adults | Yes | Yes | Yes | Yes | Yes | Yes | Yes | Yes | 8 | Low |
| Zhi, 2016  China | Associations of sleep duration and sleep quality with life satisfaction in elderly Chinese: the mediating role of depression | Yes | Yes | Yes | Yes | Yes | Yes | Yes | Yes | 8 | Low |
| Zhu, 2023  China | Reciprocal effects between sleep quality and life satisfaction in older adults: the mediating role of health status | Yes | Yes | Yes | Yes | Yes | Yes | Yes | Yes | 8 | Low |

Item 1: Were the criteria for inclusion in the sample clearly defined? Item 2: Were the study subjects and the setting described in detail? Item 3: Was the exposure measured in a valid and reliable way? Item 4: Were objective, standard criteria used for measurement of the condition? Item 5: Were confounding factors identified? Item 6: Were strategies to deal with confounding factors stated? Item 7: Were the outcomes measured in a valid and reliable way? Item 8: Was appropriate statistical analysis used?
